# Splicing complexity as a pivotal feature of alternative exons in mammalian species

**DOI:** 10.1186/s12864-023-09247-y

**Published:** 2023-04-12

**Authors:** Feiyang Zhao, Yubin Yan, Yaxi Wang, Yuan Liu, Ruolin Yang

**Affiliations:** College of Life Sciences, Northwest A&F University, Yangling, Shaanxi China

**Keywords:** Alternative splicing, Splicing complexity, Machine learning, Development and evolution

## Abstract

**Background:**

As a significant process of post-transcriptional gene expression regulation in eukaryotic cells, alternative splicing (AS) of exons greatly contributes to the complexity of the transcriptome and indirectly enriches the protein repertoires. A large number of studies have focused on the splicing inclusion of alternative exons and have revealed the roles of AS in organ development and maturation. Notably, AS takes place through a change in the relative abundance of the transcript isoforms produced by a single gene, meaning that exons can have complex splicing patterns. However, the commonly used percent spliced-in (Ψ) values only define the usage rate of exons, but lose information about the complexity of exons’ linkage pattern. To date, the extent and functional consequence of splicing complexity of alternative exons in development and evolution is poorly understood.

**Results:**

By comparing splicing complexity of exons in six tissues (brain, cerebellum, heart, liver, kidney, and testis) from six mammalian species (human, chimpanzee, gorilla, macaque, mouse, opossum) and an outgroup species (chicken), we revealed that exons with high splicing complexity are prevalent in mammals and are closely related to features of genes. Using traditional machine learning and deep learning methods, we found that the splicing complexity of exons can be moderately predicted with features derived from exons, among which length of flanking exons and splicing strength of downstream/upstream splice sites are top predictors. Comparative analysis among human, chimpanzee, gorilla, macaque, and mouse revealed that, alternative exons tend to evolve to an increased level of splicing complexity and higher tissue specificity in splicing complexity. During organ development, not only developmentally regulated exons, but also 10–15% of non-developmentally regulated exons show dynamic splicing complexity.

**Conclusions:**

Our analysis revealed that splicing complexity is an important metric to characterize the splicing dynamics of alternative exons during the development and evolution of mammals.

**Supplementary Information:**

The online version contains supplementary material available at 10.1186/s12864-023-09247-y.

## Background

Alternative splicing (AS) is an important process in gene regulation. It allows multiple mRNA transcripts to be produced from one pre-mRNA through the different combinations of splicing sites, contributing to the diversity of mature mRNA molecules in their localization, stability, and translation properties [1]. The proportion of multiexon protein-coding genes that are subjected to AS in human is up to 95%, leading to ~ 37% protein-coding genes producing more than one protein variant [2], thus greatly expanding the transcriptome and proteome repertoire [3, 4]. Typically, the splicing of exons occurs in a tissue- or development-dependent manner. A well-studied case is the titin gene, for which its long isoforms are primarily expressed in neonates, promoting passive tension of cardiomyocytes and stiffness of the myocardium wall, while the short isoforms dominate in adults [5–7]. It was observed that physiologically related splicing transitions during the development of homologous tissues/organs are largely conserved across species [8, 9]. The dysregulation of splicing site recognition leads to aberrant splicing, which can cause diseases [10].

Over the past decades, a large amount of RNA-seq data has been produced in human and other mammals, and important insights into the evolutionary and developmental dynamics of RNA splicing have been achieved [11–13]. A recent study revealed that developmentally dynamic AS events are substantially more conserved than non-dynamic ones, by assessing AS patterns across pre- and post-natal development of seven organs in mammals [14]. Many regulatory aspects of AS networks and functions have also been uncovered by focusing on the ‘percentage spliced-in’ (PSI; Ψ) values of local alternative splicing events [15]. However, AS takes place through a change in the relative abundance of the transcript isoforms produced by a single gene, which may involve complex patterns. For example, the 5’ splice site of an exon could be linked to two different 3’ splice sites, or the exon itself may have multiple 3’ splice sites and complex linkage patterns (Fig. S1A). This indicates alternative exons may differ in not only how frequently they are included in the final transcripts, but also how they are linked to multiple splice sites. If an alternative exon only involves in two transcripts (e.g., present in one transcript while absent in the other transcript), its splicing complexity is low; otherwise, we can consider the exon has high splicing complexity. Although complex splicing events can be decomposed into multiple binary events that can be quantified with the Ψ values [16, 17], such a method ignores alternative exons’ difference in linkage relationship with other exons. Recently, Sterne-Weiler et al. [18] used the Shannon’s entropy (i.e., $-{\sum }_{i}{\Psi }_{i}{log}_{2}{\Psi }_{i}$; in which ${\Psi }_{i}$ is the inclusion level of path $i$) and discrete bins of complexity [K(n); $n={log}_{2}(paths)$] to study the splicing complexity of alternative exons at the event level. Splicing entropy is a Ψ-dependent measure of AS complexity that formalizes the relative contribution of all splicing outcomes to gene expression in a read-depth independent manner. Their analysis revealed that the splicing complexity is conserved across vertebrates and elevated splicing entropy is associated with specific protein structural features [18], indicating that events with high splicing entropy have specific roles in gene function.

Furthermore, the identification of molecular mechanisms responsible for exon splicing has long been a fundamental goal in biology. Persistent efforts have revealed that whether or not an exon is included in mature mRNA is mainly determined by the recognition efficiency of the splice site by the spliceosome [19]. At the same time, RNA motifs recognized by RNA binding proteins (RBPs) and other particular RNA features/structures constitute the blueprint of the so-called “splicing code” [20], which dictates the splicing in different cell types and conditions [21–23]. Consequently, a number of in silico tools [24–27] and experimental assays [28–30] have been designed to predict AS changes at a genome-wide scale, which have shed important insights into the regulation and function of splicing code, especially in evaluating the pathogenic roles of splicing-related variants. However, these studies are all apt at predicting the Ψ values of simple AS events, there is a lack of models to predict splicing complexity of alternative exons.

Therefore, some important principles related to exons’ splicing complexity merit further investigation, such as, (i) whether exons with high splicing complexity are pervasive in mammals? (ii) what types of genes tend to contain events with high splicing complexity? (iii) can splicing complexity of exons be predicted with features of exons? (Ψ) do splicing complexity of alternative exons change among tissues and during development? (v) if so, what is the evolutionary and functional significance of changes in splicing complexity? To answer these questions, we acquired RNA-seq data from six tissues across seven species and quantified the splicing complexity of AS events (Fig. S2). Our results indicate that high-complexity events (i.e., splicing entropy ≥ 1.0; two or more splicing outcomes in a sample) are prevalent in mammals. We also revealed that the splicing complexity of exons tends to be related to features of host genes, and the machine learning and deep learning models trained using attributes of exons can moderately predict the splicing complexity of alternative exons. Additionally, we discovered that some alternative exons tend to have splicing entropy changes between tissues or during development. Overall, we systematically characterized the splicing complexity of alternative exons in mammals, suggesting that splicing complexity is another important attribute of alternative exons, in addition to the commonly used splicing inclusion level.

## Results

### High-complexity AS events are pervasive in mammalian species

**Fig. 1:**
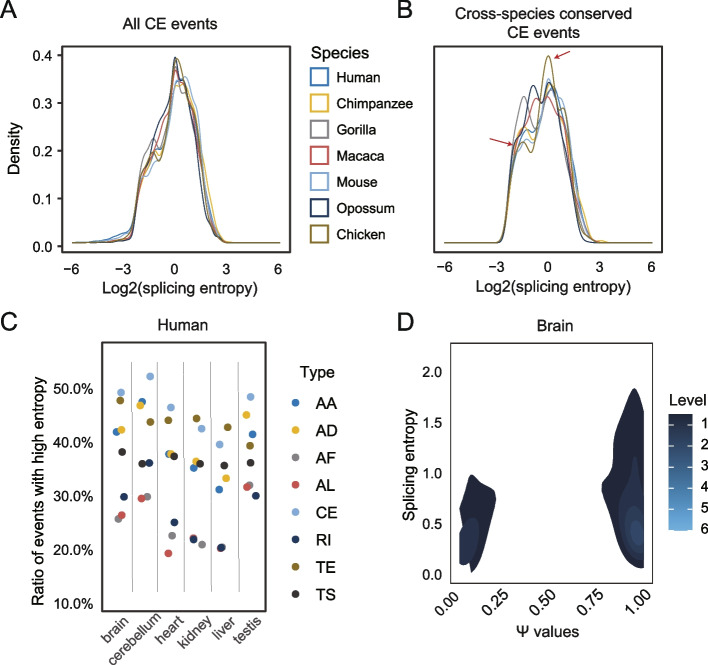
Splicing complexity of alternative exons. **A** distribution of splicing entropy for all alternative CE events in protein-coding genes in brain. **B** splicing entropy for conserved CE events across seven species in brain. Red arrows indicate the position of two peaks. **C** frequencies of events with high splicing entropy (≥ 1.0) for each type of events in human. **D** density plot for Ψ values (x-axis) and splicing entropy (y-axis) in brain of human

To investigate the pervasiveness of events with high splicing complexity, with publicly available RNA-seq data from six tissues (brain, cerebellum, heart, liver, kidney, testis) across six mammalian species (human, chimpanzee, gorilla, macaque, mouse, opossum) and chicken (Table S1) [31], we first calculated the Ψ values and splicing complexity (Ψ-dependent entropy) for all types of events from protein-coding genes expressed in each sample with Whippet [18]. It should be noted that Whippet uses the “contiguous splice graphs” (CSGs) to estimate the Ψ value for each AS event. In contrast to conventional binary events, AS events in Whippet can be of any complexity, which is defined as the collective set of skipping or connecting edges of a node (non-overlapping exonic sequences) in the CSGs (Fig. S1A). In Whippet, there are eight main types of nodes (Fig. S1B), i.e., alternative acceptor splice site (AA), alternative donor splice site (AD), alternative first exon (AF), alternative last exon (AL), core exon (CE), retained intron (RI), tandem alternative polyadenylation site (TE), tandem transcription start site (TS). The node is manifested as either a whole exon or part of an exon with flanking alternative splice sites. In this study, only nodes in expressed genes (transcripts per million, TPM ≥ 1.0) and their associated AS events with a total of at least ten supported reads were considered. Alternative exons in a sample were defined if their corresponding nodes had 0 < Ψ < 0.97; while constitutive exons were those with nodes had Ψ ≥ 0.97. As shown in Figs. 1A and S3, there are two peaks in the splicing entropy profile in all tissues, suggesting the existence of two types of exons. Notably, such a pattern is also observed for the TE, AF, AL, and TS types of AS, but is not apparent for the AA and AD types (Fig. S4). When our analysis was limited to conserved core exons in the seven species, events with low splicing entropy are more frequent (Fig. 1B). Furthermore, 20–50% of events in human show high splicing complexity (splicing entropy ≥ 1.0; i.e., two or more splicing outcomes are produced) for the eight types of events defined in Whippet. Notably, for the CE nodes, the proportion of high-complexity events is up to 40% in human brain, cerebellum, and testis (Fig. 1C). Complex splicing events are also prevalent in the other six studied species (Fig. S5). The proportion of high-complexity events is considerable even if we take splicing entropy ≥ 1.5 as a cut-off (Fig. S6). Different types of AS exhibit apparent variation in the distribution of splicing entropy and proportion of high-complexity events, which may be caused by differential usage of individual AS types in tissue. This analysis suggested that events with high-entropy account for a large proportion of AS events and make a great contribution to the complexity of transcriptome.

Next, we examined the relationship between splicing complexity (entropy) and splicing inclusion level (Ψ) of core exons in each sample. We can see from Fig. 1D that due to the high proportion of events with high Ψ values, the events with high splicing entropy appear mainly among events with high Ψ values. This pattern is observed in all species and tissues (Fig. S7). For AS types other than CE, most AS events have low splicing inclusion levels, and high-complexity events are usually found among events with low Ψ values (Fig. S8). Taken together, these results revealed that high-complexity AS events are prevalent in mammals and depicted a different perspective of AS.

To justify the robustness of the above results, we performed additional analyses. First, because splicing complexity of events are potentially affected by the completeness of transcript annotation in database, we quantified splicing entropy of the events according to annotation that were de novo assembled with RNA-seq data [32] from early organogenesis (mid-organogenesis for the heart) to adulthood across four species (human, rhesus macaque, mouse and chicken; Table S1). As a result, the proportion of CE events with high splicing complexity in each species is up to 50% in almost all species (Fig. S9). Second, sequencing depth, gene expression and splicing inclusion levels possibly influence the results of comparison, so we down-sampled total reads for each sample to the same level, or selected alternative exons in high-expression genes (TPM > 50 and total reads number > 50), or defined alternative exons with different cutoff (0.05 < Ψ < 0.95) and then repeated the above analyses. Results of these analyses show a similar pattern (Figs. S10, S11 and S12).

### Splicing complexity of exons is closely related to attributes of genes

**Fig. 2:**
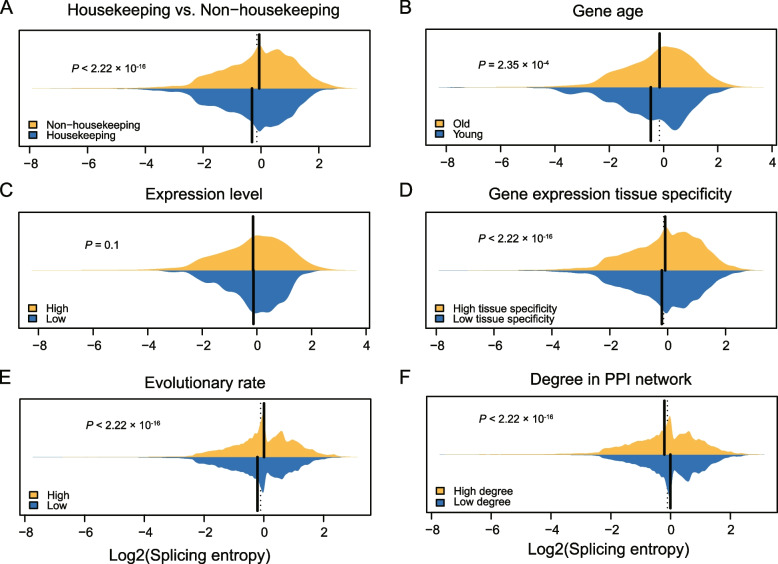
Splicing entropy of events from different types of genes. The bean charts display differential splicing entropy of AS events in genes with different categories. **A** Housekeeping genes (*n* = 16,387) vs. Non-housekeeping genes (*n* = 35,205). **B** gene age, Young: human-specific genes (*n* = 315), Old: non-human-specific genes (*n* = 40,342). **C** expression level, Low: TPM < 50 (*n* = 39,838), High: TPM ≥ 50 (*n* = 11,754). **D** gene expression tissue specificity, Low tissue specificity: tau < 0.3 (event number: 24,229), High tissue specificity: tau ≥ 0.3 (*n* = 27,363). **E** Evolutionary rate, Low (slow-evolving): 0.000923 ≤ dN/dS ≤ 0.0993 (*n* = 45,272), High (fast-evolving): 0.0993 < dN/dS ≤ 29.3 (*n* = 45,256). **F** degree in protein–protein interaction (PPI) network, Low degree: 2 ≤ degree ≤ 1.34 × 10^3^ (*n* = 25,442); High degree: 1.34 × 10^3^ ≤ degree ≤ 1.38 × 10^4^ (*n* = 25,350). All significances were evaluated with Wilcoxon rank-sum test. Dashed lines indicate the median of all two groups of CE events, solid vertical lines indicate median for each group of events

Previous research has revealed that AS events with high splicing entropy tend to reside in the intrinsically disordered regions (IDRs) of proteins [18]. We further examined what types of genes tend to contain alternative exons that have high splicing complexity. We hypothesized that alternative exons with high splicing complexity prefer to reside in some specific genes. To test our hypothesis, we limited our analysis to alternative CE events in human genes, because there is a wealth of annotated data about human genes. For example, housekeeping genes are constitutively expressed in all tissues to maintain basic cellular functions [33], we observed that events from housekeeping genes have lower splicing entropy than those from non-housekeeping genes (*P* < 2.22 × 10^–16^, two-sided Wilcoxon rank-sum test; Fig. 2A), which indicates that the housekeeping genes not only require stable transcription but also have fewer splicing variants. Interestingly, events in old genes show higher splicing entropy than those in young genes (*P* < 2.22 × 10^–6^, two-sided Wilcoxon rank-sum test; Fig. 2B), suggesting that old genes contribute disproportionately to transcriptome complexity. This is consistent with previous research which compared the splicing isoforms of genes with different ages [34, 35]. Although the splicing entropy of events in genes with different expression levels has no significant difference (*P* = 0.1, two-sided Wilcoxon rank-sum test; Fig. 2C), events in genes with high tissue specificity have significantly higher splicing entropy than those in genes with low tissue specificity (*P* < 2.22 × 10^–16^, two-sided Wilcoxon rank-sum test; Fig. 2D). Previous studies revealed that AS was associated with a considerable reduction in selection pressure on amino acid substitutions [36, 37]. We speculated that the events in genes with higher evolutionary rates may have more complex splicing patterns. As we expected, the events in fast-evolving genes show higher splicing complexity than events in slow-evolving genes (*P* < 2.22 × 10^–16^, two-sided Wilcoxon rank-sum test; Fig. 2E). And also, events in the genes with a high degree of centrality in the protein–protein interaction network show significantly lower splicing complexity than events in genes with a low degree of centrality (*P* < 2.22 × 10^–16^, two-sided Wilcoxon rank-sum test; Fig. 2F). When only CE events in genes with expression level greater than 10 TPM were considered, the same pattern was observed for all features (Fig. S13).

At last, the genes from different pathways have different splicing entropy. For example, CE events in genes from nitrogen cycle metabolic process and proteinaceous extracellular matrix have globally high splicing complexity, while events in genes associated with structural constituent of ribosome, unfolded protein binding, and vacuolar transport have low splicing complexity (Fig. S14), indicating the close association between splicing complexity of exons and gene function. In summary, these results indicate that the events with different splicing entropy are located in genes with distinct features, highlighting that splicing complexity is another important attribute of AS.

### The splicing complexity of alternative exons can be moderately predicted with machine learning

**Fig. 3:**
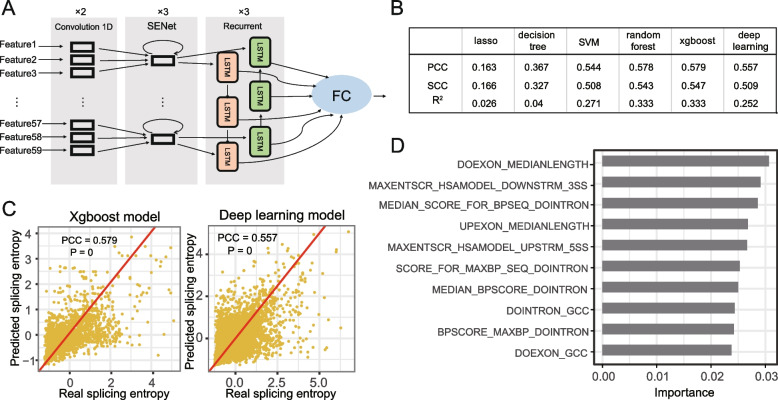
Prediction of splicing entropy with machine learning. **A** simplified diagram of deep learning model used to predict splicing entropy. For each event, 59 features were used as input and processed with two one-dimension convolutions, The subsequent Squeeze-and-Excitation Networks (SENet) was applied to process features. What follows is the recurrent layer which contains LSTM units that have end-to-end connection in both directions to capture dependencies between features. Recurrent outputs are the input of fully connected layer (FC) to predict the splicing entropy of events in test data. **B** comparison of the average performance of different methods with test data. PCC: Pearson product-moment correlation coefficient; SCC: Spearman’s rank correlation coefficient; R^2^: explained variation. **C** scatter plot shows the predictive power of xgboost and deep learning model respectively, the red line in each graph indicates the linear fit between the predicted and measured splicing entropies. **D** the rank of feature importance for the predictive splicing entropy (top 10) with xgboost model

Having revealed the importance of splicing complexity of alternative exons, we next investigated the regulatory mechanisms of splicing complexity. We hypothesized that if the splicing complexity of alternative exons is biologically relevant rather than the result of splicing noise, it could be predicted with attributes of exons. Then we focused on CE events from human protein-coding genes and extracted 59 features associated with alternative exons using Matt (see “Methods”) [38]. These features include information of the target exon, its flanking exons and introns, and splicing sites (Table S2). Considering the complexity of linkage among splice sites and the advantages of the different models in prediction, we trained different machine learning models, including lasso, decision trees, SVM, random forest, xgboost, and a deep learning (DL) model, to predict mean splicing entropy among six tissues. The architecture of the deep neural network is illustrated in Fig. 3A, in which two convolution layers are used to extract the features, three SENet layers are included to weight the feature maps to evaluate the contribution of different filters and three long short-term memory (LSTM) layers are applied to capture the interaction of different features. In general, the two nonlinear models (xgboost and deep learning) have better performance than the linear models, and the xgboost model has slightly higher accuracy than the DL model (PCC = 0.579 and 0.557, respectively; Fig. 3B, C). For both the DL and xgboost models factors contributing to the splicing pattern of exons (i.e., alternative or constitutive), such as length of flanking exons and splicing strength of upstream/downstream splice sits, are also top predictors of splicing complexity (Figs. 3D and S15). However, the contribution of individual features is limited, indicating that exon features used in the model may not be sufficient to explain complex AS events. Overall, we showed that the splicing complexity of events is moderately predictable with features associated with exons.

### Changes in splicing complexity and splicing inclusion level among tissues

**Fig. 4:**
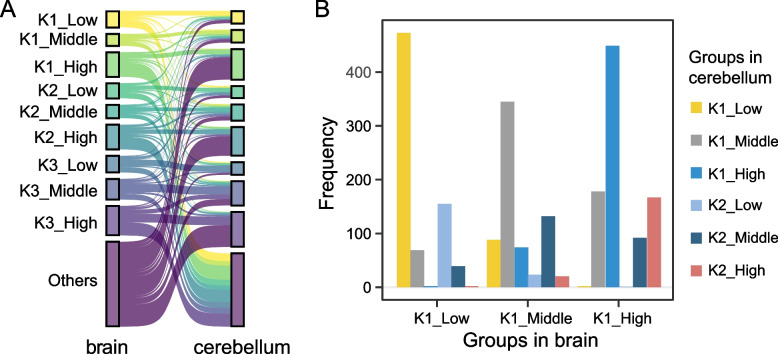
Comparison of splicing complexity and Ψ values between paired tissues in human. **A** Sankey plots show conversion of belonged groups for event among tissues. Events were categorized depending on their splicing complexity and Ψ values in each tissue, and each row is for one tissue. K{n}_{m}, n is splicing complexity category and m is Ψ category. K1_Low: complexity = K1 and 0 < Ψ ≤ 0.2; K1_Middle: complexity = K1 and 0.2 < Ψ < 0.8; K1_High: complexity = K1 and 0.8 ≤ Ψ < 0.97; K2_Low: complexity = K2 and 0 < Ψ ≤ 0.2; K2_Middle: complexity = K2 and 0.2 < Ψ < 0.8; K2_High: complexity = K2 and 0.8 ≤ Ψ < 0.97; K3_Low: complexity = K3 and 0 < Ψ ≤ 0.2; K3_Middle: complexity = K3 and 0.2 < Ψ < 0.8; K3_High: complexity = K3 and 0.8 ≤ Ψ < 0.97; Others: not in the above categories (NA in one tissue, but not NA in the other). **B** bar plot showing the frequency of events from different groups in brain and cerebellum with transcript annotation from Ensembl database

Previous research revealed that some alternative exons function in a tissue-specific manner, by comparing Ψ values of each event among tissues [4, 39]. However, few studies have investigated splicing complexity changes of alternative exons among tissues, and little is known about the contribution of splicing complexity to the splicing changes of exons in different tissues. To this end, we compared AS change among human tissues at both the splicing inclusion and splicing complexity levels. Owing to the inner correlation between splicing complexity and splicing inclusion level, the two features were investigated and compared simultaneously. To simplify the comparison, events having Ψ = 0 or with not enough reads to quantify in both compared tissues were excluded. The CE events were classified into ten classes (Fig. 4A), depending on discrete bins of splicing complexity (Kn) and Ψ values. For example, K1_Low refers to events that have simple splicing pattern (complexity = K1; produce at most two splicing outcomes) and have a low Ψ value (0 < Ψ ≤ 0.2). Globally, only a few events belong to same class in any two compared tissues (Figs. 4A and S16), revealing the huge difference between tissues in both splicing complexity and splicing inclusion level. Considering the AS events in brain and cerebellum, only about one-third of events from class K1_Low in brain are still belong to K1_Low in cerebellum (Fig. 4A), representing events that have minor changes in both splicing complexity and splicing inclusion level. Most of the remaining events from class K1_Low in brain were categorized into the “others” class (i.e., exons with no mapped reads) in cerebellum. Among the K1_Low events in brain, those categorized into the K2_Low class (change only in splicing complexity) were almost twice as many as those categorized into the K1_Middle class (change only in splicing inclusion level) in the cerebellum (Fig. 4B), which indicates that a larger proportion of events changed in splicing complexity between the two tissues compared with that in splicing inclusion level. To mitigate the influence of difference in sequencing depth and completeness of transcript annotation, down-sampled bam files or de novo assembled transcripts were used to repeat the analysis. As a result, the same pattern is observed (Figs. S17 and S18), supporting the robustness of our analyses. Our analysis revealed that changes in splicing entropy and splicing inclusion level have a comparable contribution to tissue-specific splicing difference.

As a case, exon 16 (2:165,327,155–165,327,202) of the *SCN2A* gene has similar Ψ values but different splicing complexity between brain and cerebellum. Specifically, there are two splicing patterns for this exon in cerebellum, and only one splicing pattern in brain (Fig. S19A). On the other hand, exon 2 (1:159,189,771–159,189,872) of the *CADM3* gene has two splicing patterns in brain and cerebellum, but the Ψ values are substantially different (0.112 vs. 0.271) in the two tissues (Fig. S19B).

### Evolutionarily old AS events have high splicing entropy in mammalian clade

Valuable insights have been obtained by comparing the Ψ values of events with different splicing ages [11, 12]. We asked whether the evolutionary dynamics of splicing complexity could also be used to reveal the evolutionary rules of alternative exons. To this end, exons with different splicing ages in human were compared. For simplicity, the following comparative analysis was limited to CE nodes that had one-to-one orthologs across all the seven species and were alternatively spliced in at least one of the studied species. Cluster analysis based on the Ψ values of 22,294 orthologous exons (in 5,255 one-to-one orthologous genes) recapitulated the previous findings that the splicing of exons presented a species-dominated clustering pattern, i.e., the exons from different tissues within species showed more similar Ψ values than that of the matching tissues among different species (Fig. S20A). When clustering using splicing entropy of the same set of CE events, a similar pattern was obtained (Fig. S20B). What’s more, the clustering result using Ψ values of 251 CE nodes (in 204 one-to-one orthologous genes) that are conservatively spliced in all the seven species presented a prominent tissue signature (Fig. S20C), consistent with the findings of Merkin et al. (2012) and Barbosa-Morais et al. (2012) [11, 12]. However, the result based on splicing entropy of the same set of conservatively spliced exons showed slightly inconsistence compared with that based on splicing inclusion level (Fig. S20D). It can be observed that although the brain and cerebellum are clustered together and separated from other tissues, the samples of primates are also separated from non-primate samples, possibly because splicing complexity evolves faster than splicing inclusion level. Together, these results suggested that splicing entropy could be used to characterize the evolution of alternative exons.

**Fig. 5:**
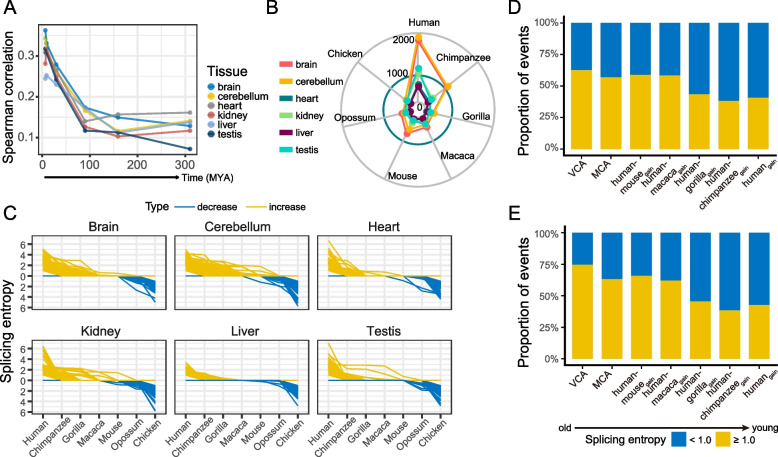
Evolution of splicing complexity. **A** Spearman correlation between human and other species when comparing splicing entropy pairwise for orthologous events in each tissue. For each pair of species, correlation is calculated for splicing entropy of all orthologous exons in the seven species. **B** the numbers of conserved alternative exons in seven species with highest splicing entropy in each species for each tissue. **C** line chart showing splicing entropy of events with decrease/increase in splicing entropy during evolution in each tissue. **D** bar plot displaying the ratio of alternative exons with maximum splicing entropy ≥ 1.0 for each group. **E** bar plot displaying the ratio of alternative exons with maximum changes in splicing entropy ≥ 1.0 among tissues for each age group of events. VCA: vertebrate conserved alternative exons; MCA: mammalian conserved alternative exons

We next examined the relative rate at which splicing entropy has evolved. By pairwise comparisons of splicing entropy in homologous tissues between human and the other six species, we observed that splicing entropy similarity to human decreased with evolutionary divergence from human for each tissue (Fig. 5A). Then, we inspected in which species the orthologous events tended to have the highest splicing entropy. We investigated those events for which the difference between the top two splicing entropy among the seven species was up to 1.0, and found that CE events with highest splicing entropy frequently occur in human (Fig. 5B). This pattern is observed in all the six tissues, suggesting that AS events in human tissues tend to have the highest splicing complexity. Then, we extracted events that showed monotonic changes in splicing entropy during evolution for each tissue (Fig. 5C). Among these events, more than half of events showed increased splicing entropy during evolution. However, when concerning AS events that showed evolutionarily consistent change in splicing entropy across the six tissues, we found that there are a few overlaps among tissues (Fig. S21), revealing the evolutionary difference of AS events in splicing complexity among tissues. Furthermore, we examined whether the genes containing events with decreased-complexity have different functions compared with those having events with increased -complexity. Gene ontology enrichment analysis revealed that genes containing events with monotonic increased or decreased splicing complexity are related to different terms. For example, in brain, genes containing events with increased complexity are enriched in delayed speech, language development and cell leading edge, while genes containing events with decreased complexity are enriched in some complex formation (Fig. S22).

By fixation of mutations that affect splicing, constitutive exons are an evolutionary source of new alternative exons, a process that was termed as “alternification” [40]. Thus, we assessed the evolutionary change of exons in splicing entropy after “alternification”. A total of 36,731 one-to-one orthologous CE events with estimated Ψ values in all seven species were analyzed. These exons were classified into different categories based on the time of splicing state changes along the phylogeny of the seven species. The estimation of splicing age was performed using parsimony, following the method of Merkin et al. (2012) [12] (Fig. S23; see Methods). By comparing the maximum entropy of alternative exons among the six tissues, we found that the ratio of high-complexity exons (entropy > 1.0) generally increased with their splicing ages (Fig. 5D). For example, the proportion of high-complexity events for human-specific alternative exons (human_gain_) is 38.5%, while this number is doubled (74.5%) for vertebrate conserved alternative exons (VCA). Meanwhile, a large proportion of events present substantial difference (entropy change > 1.0) in splicing entropy among tissues (Fig. 5E; 38.1% in human_gain_, 62.5% in VCA), highlighting the importance of change in splicing complexity during organ evolution. It should be noted that the above estimates are conservative because splicing entropy < 1.0 can also occur for events with more than two expressed isoforms. These results suggest that at the onset of the switch from a constitutive exon to an alternative exon, the alternative exon usually exhibits low splicing complexity, then evolve to be an alternative exon with higher splicing complexity.

### Alternative exons are combinatorically regulated by splicing complexity and splicing inclusion level during development

**Fig. 6:**
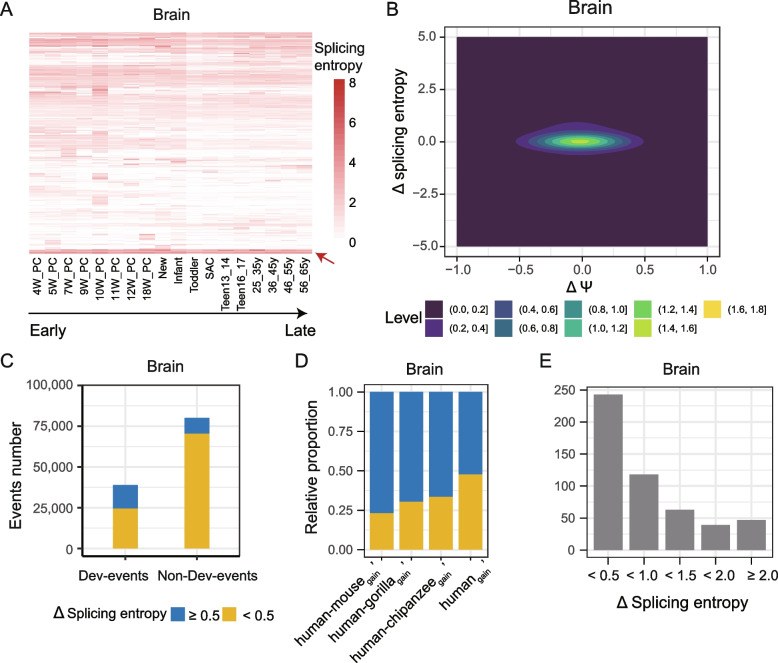
Splicing complexity changes during development. **A** hierarchical clustering for splicing complexity for events that have confident change (at least 20 supported reads) in splicing entropy larger than 0.5 and expression level of located gene larger than 10 TPM. Red arrow represents events that have stable and high splicing entropy during development. **B** 2D kernel density plots showing the largest change in splicing entropy (y-axis) and Ψ (x-axis). **C** the number of events that change in splicing entropy. Dev-events: events with largest splicing entropy ≥ 0.5 during development, non-Dev-events: events with largest splicing entropy < 0.5. **D** The relative ratio of events with splicing entropy changes larger than 0.5 among events that are not regulated in Ψ (max Δ Ψ < 0.1). **E** the distribution of splicing entropy changes for events in the human_gain_’ group

We have shown the splicing complexity of alternative exons change frequently among tissues. AS may also play essential roles during organ development [1, 15]. However, most research about AS were limited to the study of adult tissues. To this end, we analyzed the RNA-seq data of six human tissues (brain, cerebellum, heart, liver, kidney, and testis) from early organogenesis to adult (Fig. S24; Table S1) [32]. To explore the splicing complexity change during development, CE events were deliberately selected for each tisssue so that all events had splicing entropy change ≥ 0.5, had more than 20 supported reads, and their host genes’ expression level were larger than 10 TPM in at least five developmental stages. Cluster analysis revealed that some AS events have stable and high splicing complexity, while other events show stage-specific high splicing entropy during development (Figs. 6A and S25), revealing dynamic changes of splicing complexity between developmental stages. For example, exon 26 (12:56,318,237–56,318,434) in the *PAN2* gene has low splicing complexity (splicing entropy = 0.68) at 4th week post-conception, but has high splicing complexity at SAC (7–9 years old) and adult stage (splicing entropy = 2.03 in both tissues; Fig. S26). At the transcript level, an additional isoform that includes the target exon while skips the upstream exon appears at SAC and aldult stages, but is absent at 4th weeks post-conception. However, little change in Ψ values (< 0.1) is observed among these three stages for this exon (Fig. S26).

Because splicing inclusion level and splicing complexity are correlated to some extend, we examined the distribution of splicing inclusion level and splicing entropy changes for developmentally regulated events (Dev-events, ΔΨ ≥ 0.1 relative to 5th weeks post-conception for testis, 4th weeks post-conception for other tissues). Figure 6B shows the maximum change in splicing entropy and Ψ values in brain, which exhibits symmetric distribution centered on zero. This result indicates that for different AS events, changes of splicing inclusion level and splicing complexity are not linearly correlated during development. Interestingly, it can be observed that a bump appears in the density plot of each tissue, except for the heart (Figs. 6B and S27), which suggests that some AS events show excessive increase in splicing entropy during development. We further compared changes of splicing entropy for Dev-events and non-Dev-events (ΔΨ < 0.1 relative to 5th weeks post-conception for testis, 4th weeks post-conception for other tissues) during development. We found that 10–15% of events have negligible change in splicing inclusion level (i.e., non-Dev-events) but show a splicing entropy change larger than 0.5 (Figs. 6C and S28). It should be noted that the proportion of events showing splicing entropy change is larger for Dev-events than that for non-Dev-events (Figs. 6C and S28). Overall, our analysis revealed that splicing inclusion level and splicing entropy cooperate together to characterize AS events’ dynamics during development.

Furthermore, to investigate the evolutionary dynamics of Dev-events, we compared splicing complexity during development for exons with different splicing age. To obtain enough events for comparison, we limited our analysis to exons that are conserved across five mammalian species (human, chimpanzee, gorilla, macaque, mouse), and classified these exons into different age groups based on the time of splicing state changes along the phylogenetic tree of the five species. For almost all age groups, more than half of non-Dev-events exhibit substantial splicing entropy changes in each tissue (Fig. S29A). Meanwhile, for the brain tissue, the proportion of AS event exhibiting apparent complexity change among non-Dev-events shows a monotonic increase with the phylogenetic breadth of alternative exons (Fig. 6D). Among human-specific non-Dev-events, 22.9% (liver) to 33.7% (heart) alternative exons have maximum changes of splicing entropy larger than 0.5 during organ development (Figs. 6E and S29B). Gene Ontology (GO) enrichment for genes containing non-Dev-events in the human_gain_^’^ (human-specific CE) group revealed that the top enriched terms are similar for events with low and high splicing entropy change (Fig. S30), such as ﻿autophagy and ﻿nuclear envelope. In addition, genes containing events with low splicing entropy change are also specifically enriched in ﻿cellular amino acid metabolic process, ﻿plasma membrane organization, ﻿cytoskeleton − dependent intracellular transport, and ﻿cell junction organization. Overall, our analysis indicates that evolutionarily old alternative exons contribute more to the diversity of transcriptome, and that splicing changes during development are the result of combinatory regulation of exons in inclusion level and splicing complexity.

## Discussion

A fundamental question in biology is to decipher and understand genotype–phenotype map. Alternative splicing, a basic biological process in eukaryotes, provides an essential layer of gene expression regulation that directly influence cell identity and cell fate. Therefore, characterizing the genome-wide and tissue-wide AS pattern of exons and their evolutionary history becomes the first and most important step in deciphering the genetic features responsible for particular AS events and the dynamics of AS. As the basic unit of transcript, exons have specific advantage in gene function evolution. Due to lower selective pressure compared with the birth of new exons or genes, the creation of new transcript by exons’ combination without altering genome sequence and structure is a shortcut to produce new functional proteins during evolution, although whether most of the isoform diversity arising from AS represents transcriptional noise has been debated [41–43]. What has been confirmed is that alternative exons tend to be interaction domain and rewire tissue-specific protein–protein interaction network [1, 17, 44, 45]. The frequent involvement of exons in assembly of different transcript creates not only a large number of alternative exons, but also exons with complex splicing pattern in tissues (Fig. 1C).

To date, tissue-specific or developmentally regulated AS is mainly limited to the quantification and comparison of changes in splicing inclusion level of exons with short reads [14, 46–49]. Splicing inclusion level of alternative exons evaluates change in exon usage, but it overlooks change of the exon’s connectivity with other splice sites. Ideally, the splicing complexity of exons can be quantified by comparing the number of splicing isoforms and the expression level of all splicing isoforms. Therefore, some efforts have been dedicated to develop specialized protocols to infer full-length mRNA isoforms with short reads. For example, Tilgner et al. [50] developed a “synthetic long read” RNA-seq approach for isoform assembly with next-generation sequencing data. However, this method is also limited by the error-prone nature of de novo assembly with short reads. Moreover, the assumption that one RNA molecule per gene in each pool might not hold true for highly expressed genes. Continuing interest in sequencing full-length mRNA transcripts also promote the prosperity of third generation sequencing technology that can determine exon connectivity and even full-length mRNAs, especially for genes with complex AS pattern or with thousands of distinct isoforms [51, 52]. Recently, Glinos et al. [53] used long reads to compare the tissue-specificity of transcript structure, and found that cerebellar hemisphere, liver and fibroblasts have the highest ratio of tissue-specific transcript [53]. But due to its low throughput, third generation sequencing can only provide a relatively small number of reads, leading to an inaccurate quantification of the relative isoform expression for complex spliced genes. What’s more, the large sequencing expense of this platform can also be a prohibitive barrier to many researchers. Thus, it remains difficult to accurately and directly determine the connectivity of exons within the same transcript at the genomic scale. However, as with what we found in this research, a large number of exons have high splicing complexity (Fig. 1C) and the splicing complexity difference reflects splicing change among tissues (Fig. 4A). So, splicing entropy used in the present study is a tentative yet indispensable metric to leverage the study of splicing complexity of exons with short-read high-throughput RNA-seq data.

Interestingly, the linkage complexity of exons is closely related to gene age, evolutionary rate, gene function, and gene connectivity in interaction network. Although the causal relationship still needs further research, the correlation between splicing complexity and gene feature exists. This reveals that the splicing complexity of exons is not randomly distributed among genes, but plays a significant role in genes from specific pathways (Fig. 2A-F).

Mechanically, we revealed that splicing complexity of alternative exons were partly controlled by exons' features, including length of flanking exons and splicing strength of downstream/upstream splice sites (Fig. 3D). The accuracy of splicing complexity prediction is relatively low compared with that of splicing inclusion level prediction by deep learning models [27]. The underlying reasons may include three aspects: first, splicing complexity of exons are essentially more complicated, because the splicing complexity is potentially involved in the combination of all potential splice sites of a gene, while whether GT/AG sequence is a splicing site is mainly decided by local sequence feature; second, because of the lack of tissue information in our prediction variables, we can’t predict the Ψ values variation among tissues; third, the architecture of the model needs further optimization in future research. Fortunately, with the continuing optimization of machine learning constructure and increasingly available computing source, it is promising to predict the linkage complexity of exons in tissues from DNA/RNA sequences directly.

The control of linkage pattern of exons can be created by molecular-level changes acting on developmental programs related to tissue functions and morphologies, providing a foundation for lineage-specific innovation [54]. Splicing complexity across species can also be used to uncover the evolutionary dynamics of alternative exons (Fig. S20). Previous research has revealed that old alternative exons have low inclusion level, high splicing tissue specificity along with high evolutionary rate by comparing Ψ values [12]. Accordingly, this study showed old alternative exons tended to have high splicing complexity and large splicing entropy variation among tissues (Fig. 5D, E). This revealed that the exons might change in splicing complexity after evolving to be an alternative exon from a constitutive one, suggesting organisms frequently regulated isoforms expression by the transition of exons’ connectivity, in addition to the usage rate of exons.

Phenotypically relevant AS change may occur during embryonic development, while the majority of comparative transcriptomic studies about AS have been focusing on splicing inclusion level (Ψ) of alternative exons, especially in adult organs [48, 49]. A recent relevant study comparing AS changes in splicing inclusion levels across pre- and postnatal development of seven organs in seven species [14]. In that paper, the authors analyzed the proportion of development-regulated events in exons with different splicing ages, also revealed that developmentally dynamic AS events are more conserved than non-dynamic ones [14]. Here, we revealed that the developmentally dynamic splicing change occurred not only in Ψ values but also in splicing complexity (Fig. 6C, D). However, an important limitation of our analysis is that bulk RNA-seq don’t allow assess the relative contribution of cellular composition change versus cell-type intrinsic splicing complexity change to observed change in splicing complexity during development. It requires single-cell RNA-seq data that allows reliable quantification of the splicing complexity in different cell types to disentangle the contributions. At last, the contribution of splicing complexity change to phenotypic change during development still needs further functional studies.

## Conclusions

In summary, our work revealed the roles of splicing complexity in characterizing the diversity for exons’ connectivity among tissues and developmental dynamics. Although the splicing complexity of exons is not a perfect solution to disentangle the precise contribution of each isoform to gene expression level and phenotypic changes, it is a compromise way to combine splicing complexity and Ψ values before third-generation sequencing technology is mature enough to quantify expression of each isoform.

## Methods

### Data collection

The 76-bp and 101-bp RNA-seq datasets from brain, cerebellum, heart, kidney, liver, and testis of 7 species: human (*Homo sapiens*), macaque (*Macaca mulatta*), mouse (*Mus musculus*), opossum (*Monodelphis domestica*), chimpanzee (*Pan troglodytes*), gorilla (Gorilla gorilla) and chicken (*Gallus gallus*) were downloaded from Gene Expression Omnibus (GEO accession ID: GSE30352). The reference genomes and annotation files (gene transfer format, GTF) were retrieved from Ensembl annotations, release 92. The information of protein-coding genes and longest protein-coding transcripts were extracted from GTF files.

### RNA-seq data preprocessing

The quality of reads was controlled using FastQC (http://www.bioinformatics.babraham.ac.uk/projects/fastqc/). To obtain a homogeneous comparison, the samples were treated as follows: 1) either the single-end reads or the forward reads of the paired-end reads were used, 2) the reads of all samples were trimmed to 70 bp, and 3) reads with more than 5 N bases were filtered out using Trimmomatic-0.36 [55]. At last, reads from samples of the same tissue were combined for further analysis.

### Gene expression and AS quantification

We used Whippet (v0.5) [18] to detect and quantify Ψ and splicing complexity of AS events. First, the RNA-seq reads were mapped onto the respective reference genome with TopHat2 (v2.1.1, -N 3, –no-novel-indels, –no-discordant, –no-mixed) [56]. The unique reads from mapped bam files for the same species were merged and sorted, and duplicates were removed with SAMtools v1.4.1. In order to identify new transcripts, we ran Cufflinks (version 2.2.1, -I 200,000 –max-bundle-length 10,000,000) on merged reads. Cuffcompare was used to compare predicted transcripts with that of annotated GTF file from the Ensembl database. Transcripts with Cufflinks class codes of “c”, “j”, “ = ”, “e”, or “o” were kept for further analysis. The merged bam file for each species and the derived GTF files were combined to create splice graphs. Whippets was run using the default settings to quantify events and gene expression levels for each sample. In brief, annotated transcripts from genes were collapsed into non-overlapping exon bins (nodes), then a Contiguous Splice Graph (CSG) was built. In CSG, edges represent splice junctions or adjacent exonic regions, so that isoforms can be represented as paths through nodes. After aligning to edges, paths through each node’s AS event can be estimated. For each exon, all paths through the AS event are enumerated and quantified. The Ψ value of a node is defined as the sum of the relative abundance of the paths containing the node [18] (Fig. S1A). Splicing complexity of exon can be classified into discrete bins of complexity based on the total enumerated paths number from the event (n = log2(path number), such that K(n) can produce at most 2^n^ spliced outcomes), or Ψ-dependent Shannon’s entropy (entropy = -${\sum }_{i}{\Psi }_{i}{log}_{2}{\Psi }_{i}$, ${\Psi }_{i}$ is the inclusion level of path *i*). The two metrics are related, because the maximum entropy for an event with K(n) is n according to above formula [18]. Only nodes in expressed genes (TPM ≥ 1) and with a total of at least ten supported reads for the associated AS events were kept. To estimate the bias from annotation completeness, we obtained the de novo transcript annotation from (https://apps.kaessmannlab.org/alternative-splicing/) which performed detailed de novo annotation of transcribed regions for all species by RNA-seq data of (forebrain/cerebrum, hindbrain/cerebellum, heart, kidney, liver, ovary, and testis) from early organogenesis (mid-organogenesis for the heart) to adulthood across six mammals. These annotated transcripts from four species (human, rhesus macaque, mouse and chicken) were used in this study to alleviate biases from genome annotation quality differences among species. The same quantification process was used to calculate splicing complexity. To mitigate the effect of sequencing depth, each sample were down sampled to the same level (Chicken brain: 37,033,946 sequencing reads) and then quantified the splicing complexity. At last, events with sufficient supported reads (reads number ≥ 50) in genes with high expression (TPM ≥ 10) were used to compare splicing entropy.

### Gene sets with different features

Housekeeping gene list (3,804) was obtained from Eisenberg et al. (2013) [33], and other protein-coding genes not in the list were considered as non-housekeeping genes. The genes were classified into two groups: Low expression (TPM < 50) and High expression (TPM ≥ 50). Tissue specificity was measured across six tissues using the metric tau [57]:

$$\tau =\frac{{\sum }_{i=1}^{n}(1-\widehat{{x}_{i}})}{n-1}; \widehat{{x}_{i}}=\frac{{x}_{i}}{\underset{1\le i\le n}{\mathrm{max}}{x}_{i}}$$

${x}_{i}$ is the expression of the gene in tissue $i$, $n$ is the number of tissues.

Genes were classified into low tissue specificity (tau < 0.3) and high tissue specificity (tau ≥ 0.3). Gene age data were retrieved from Yin et al. (2016) [58]. Similarly, the genes were classified into Young (human-specific) and Old (non-human specific). dN and dS for each protein-coding gene were retrieved from Ensembl v92, and genes were classified into two groups (slow-evolving: 0.000923 < dN/dS < 0.0993 and fast-evolving: 0.0993 < dN/dS < 29.3) with equal gene number. Protein–protein interaction networks for human were downloaded from the string database (https://cn.string-db.org). This network included 19,385 nodes and 11,938,498 edges. Degree for each gene was calculated with the function “degree” in the R package igraph [59]. Wilcoxon rank-sum test was used to compare differences in splicing entropy of events from genes with different features.

### Identification of orthologous exons

One-to-one orthologous protein-coding genes were obtained from Ensembl release 92. We used two sets of orthologous genes: 9371 orthologous genes for all seven species (all studied mammalian species and chicken as an outgroup) and 13,442 orthologous genes for five species (human, chimpanzee, gorilla, macaque, mouse). For each orthologous gene, the exons of the longest protein-coding transcript were used to identify orthologous exons. The orthologous exons that were used for cross-species comparison of alternative splicing events were obtained by converting the coordinates of the exons using LiftOver from UCSC with at least 0.75 match (-minMatch 0.75) according to the respective all-chain BLASTZ file. Only one-to-one orthologous exons were used for further analysis in all considered species. The first and last exons in all transcripts were abandoned. Finally, 69,033 and 117,374 exons were obtained for the two sets of genes.

### Estimation of the ages of alternative splicing events

The phylogenetic tree for the seven species was retrieved from the TimeTree database (http://www.timetree.org/). The one-to-one orthologous exons with CE (“core exon”) events defined in Whippet were considered to be orthologous CE events. At last, 47,592 and 83,172 orthologous CE events were obtained for the two sets of genes. For simplicity, we considered ‘exonic bins’ in Whippet to be ‘exons’ throughout the text.

To facilitate the comparison of exon splicing patterns in various tissues, we classified exons into two major groups, according to gene expression level and Ψ values in one tissue: 1) alternative CE events, i.e., CE events (0 < Ψ < 0.97) in expressed genes (TPM ≥ 1) and with a total of at least ten supported reads for the associated AS event; 2) constitutive CE events, i.e., CE events (Ψ ≥ 0.97) in expressed genes (TPM ≥ 1) and with a total of at least ten supported reads for the associated AS event.

One tissue or all six tissues can be used when identifying the splicing pattern of one exon in a species. Alternative CE event were defined as those with associated exons that are alternatively spliced in at least one of the six tissues, while constitutive CE events are those with the associated exons that are constitutively included in all quantifiable tissues. Orthologous CE events that were neither identified as alternative CE events nor constitutive CE events in any species were not used for further analysis.

After identifying the splicing pattern of orthologous CE, exons were defined in each species according to their splicing patterns in one tissue or all tissues, orthologous alternative CE events occurring in a subset of species could be considered as evolutionary gain (constitutive to alternative spliced) or loss (alternative spliced to constitutive) of alternative splicing. Parsimonious principle was applied to identify the gain and loss of AS and its evolutionary age, following the method described in Merkin et al. (2012) [12]. The exons that could be explained by one gain or loss event were grouped to different splicing age classes according to the time of gain or loss, the cases that could not be explained by one gain or loss event were grouped into the “complex” group (Fig. S23). For example, if the splicing pattern for an exon in seven species can be explained by an evolutionary gain of skipping in the most recent common ancestor of human and opossum, then the exons were grouped into the human-opossum_gain_ group. Orthologous CE events that were constitutive/alternative exons in all studied seven species were grouped into VCC (vertebrate conserved constitutive exons) or VCA (vertebrate conserved alternative exons). Exons that were constitutive in chicken and alternative exons in all mammalian species were grouped into MCA (mammalian conserved alternative exons). If the opposite is true, the exons were grouped into MCC (mammalian conserved constitutive exons). For exon groups from the five-species phylogeny tree, the group names were additionally labeled with a single quotation mark. For example, considering an orthologous CE event that is alternative in human and chimpanzee and constitutive in gorilla, macaque and mice, the exons were grouped into human-chimpanzee_gain_’.

Approximately half of the analyzed exons (17,687) are constitutive across the seven species (vertebrate-conserved constitutive exons, VCC); 1,305 exons are constitutive in the mammalian lineage but alternatively spliced in chicken (mammal conserved constitutive exons, MCC); 251 exons are alternatively spliced in all seven species (vertebrate-conserved alternative exons, VCA); and 130 are alternatively spliced in mammals but constitutive in chicken (mammal conserved alternative exons, MCA). Given the present phylogeny, MCA contains two types of exons, corresponding to the gain of AS in the mammalian lineage or the loss of AS in chicken, respectively. We also identified several sets of exons for which the directions and the ages of AS gains or losses can be reliably inferred (Fig. S23). For example, there are 170 AS gains in the lineage before the divergence between the mouse and primates, 487 AS gains in the primate lineage, and 1,532 AS gains in a human-specific manner (Fig. S23). Species-specific gain of AS is also pervasive in other mammalian species (1,446 in chimpanzees, 982 in gorillas, 1,525 in macaques, 1,318 in mice, and 1,619 in opossum). Compared to AS gain, AS losses are much rarer during mammalian evolution, with only 239 losses of AS with varying evolutionary ages being detected (Fig. S23). Additionally, 6,824 exons experienced more than one-time changes in the AS state in the phylogeny, which were assigned to the “complex” group (Fig. S23).

### Gene Ontology and KEGG enrichment analysis

The GO slim annotation for human was achieved from bioMart of Ensembl release 92. The genes with more than two exons in their longest protein-coding transcripts were used as the background gene set, and Gene Ontology and KEGG pathway [60] enrichment analyses were performed using g:Profiler (https://biit.cs.ut.ee/gprofiler/gost), which uses a hypergeometric test with Bonferroni correction.

### Model building for splicing entropy

The coordinates of CE events were extract from Whippet output, and the get_efeatures command of *Matt* [38] was used to extract features with the GTF file of human. At last, 59 features for each exon were fetched, including attributes of the target exon (e.g., length, GC content, exon rank in transcript), attributes of its upstream and downstream exons and introns (e.g., length, GC content, pyrimidine content), information of splicing sites (e.g., strength, maximum entropy score of 3’ splice-site and 5’ splice-site). Table S2 gives a detailed description of all features. The extracted features were used as predictor variables and splicing entropy as response variable to train five machine learning models respectively, including multiple linear regression, decision tree, random forest, xgboost, and SVM. The hyperparameters grid were created using the grid_regular function in the R packages dials and tuned with fivefold cross-validation on training data with the tune_grid function in the R package tune. The hyperparameters with the lowest error rate were used in modeling. The performance of each model was evaluated by the Pearson product-moment correlation coefficient (PCC), Spearman correlation coefficient (SCC) and the relative contribution to R^2^ on test data. The importance of predictors was evaluated with the vi function in the vip package.

### Deep learning model built for splicing entropy

As illustrated in Fig. 3A, our model architecture utilized SENet (Squeeze-and-excitation networks) [61] to weight the feature maps to evaluate the contribution of different filters, and utilized long short-term memory (LSTM) to allow the interaction of different features. All data were divided into the training set, validation set, and test set by 8:1:1. Our models are trained using the Adam algorithm as an optimizer and a minibatch size of 10,000 to minimize the mean squared error (MSE) on the training set. Validation loss was evaluated at the end of each training batch and stopped during the training procedure since we found the loss curve of validation data showed a sign of plateauing. The importance of features was evaluated with Gradient-weighted Class Activation Map (Grad-CAM) algorithms after the training step (Grad-cam: Visual explanations from deep networks via gradient-based localization). The deep learning model was written in Python 3.8.8, utilizing the PyTorch 1.7.0 library, and was trained on a Linux server with NVIDIA Titan X Pascal GPU.

### Analysis of RNA-seq time-series data

The RNA-seq datasets of six major organs (brain, cerebellum, testis, heart, liver, and kidney) in human were obtained from a Cardoso-Moreira et al. (2019) (Table S1; [32]). This dataset includes prenatal development from 4 to 20 weeks post-conception, postnatal development from neonates to teenagers and adults from 20 to 63 years. Postnatal development was grouped into neonates (New; unit: days), infants (Infant: 6–9 months; unit: months), toddlers (Toddler: 2–4 years; unit: years), school (SAC: 7–9 years; unit: years) and teenagers (Teen: 13–19 years; unit: years). For quantification, Whippet (v0.5) was run using the previously created index files with default parameters. Whippet’s probability estimate was used to identify differentially spliced alternative exons by comparing the first stage (5th weeks post-conception for testis, 4th weeks post-conception for other tissues) with other stages. AS events with |ΔΨ| ≥ 0.1, posterior possibility ≥ 0.85 were considered to be differentially spliced events. Exons were defined as developmental regulatory events (Dev-events), when they were identified as differentially spliced exons in at least five stages compared with the first stage, otherwise, events were defined as non-Dev-events.

### Data analysis

All statistical analyses and plots were done in R (version 3.56). Plots were created using the ggplot2 [62].

## Supplementary Information

**Additional file 1.**

**Additional file 2.**

**Additional file 3.**

## Data Availability

The information about the datasets used and/or analyzed is available in the manuscript or the supplementary materials.

## Abbreviations

AA: Alternative acceptor splice site
AD: Alternative donor splice site
AF: Alternative first exon
AL: Alternative last exon
AS: Alternative splicing
CE: Core exon
CSGs: Contiguous splice graphs
Dev-events: Developmentally regulated events
GO: Gene Ontology
Grad-CAM: Gradient-weighted Class Activation Map
IDRs: Intrinsically disordered regions
LSTM: Long short-term memory
MCA: Mammalian conserved alternative exons
MCC: Mammalian conserved constitutive exons
MSE: Mean squared error
non-Dev-events: Non-developmentally regulated events
PPI: Protein–protein interaction
RBPs: RNA binding proteins
RI: Retained intron
SCC: Spearman correlation coefficient
SENet: Squeeze-and-excitation network
SVM: Support vector machine
TE: Tandem alternative polyadenylation site
TPM: Transcripts per million
TS: Tandem transcription start site
VCA: Vertebrate conserved alternative exons
VCC: Vertebrate conserved constitutive exons
