# Supplementary material for: Splicing complexity as a pivotal feature of alternative exons in mammalian species
Source: BMC Genomics. 2023 Apr 12;24:198. doi: 10.1186/s12864-023-09247-y (PMC10099729; doi:10.1186/s12864-023-09247-y)
Supplement: Supplementary file 3 — Additional file 3. [file 12864_2023_9247_MOESM3_ESM.docx]

## Supplementary Material

**Supplemental tables**

Table S1 Summary of publicly available datasets used in paper.

Table S2 Description of features (75 and 59 features) used in splicing entropy prediction by machine leaning models.

**Supplemental Figures**


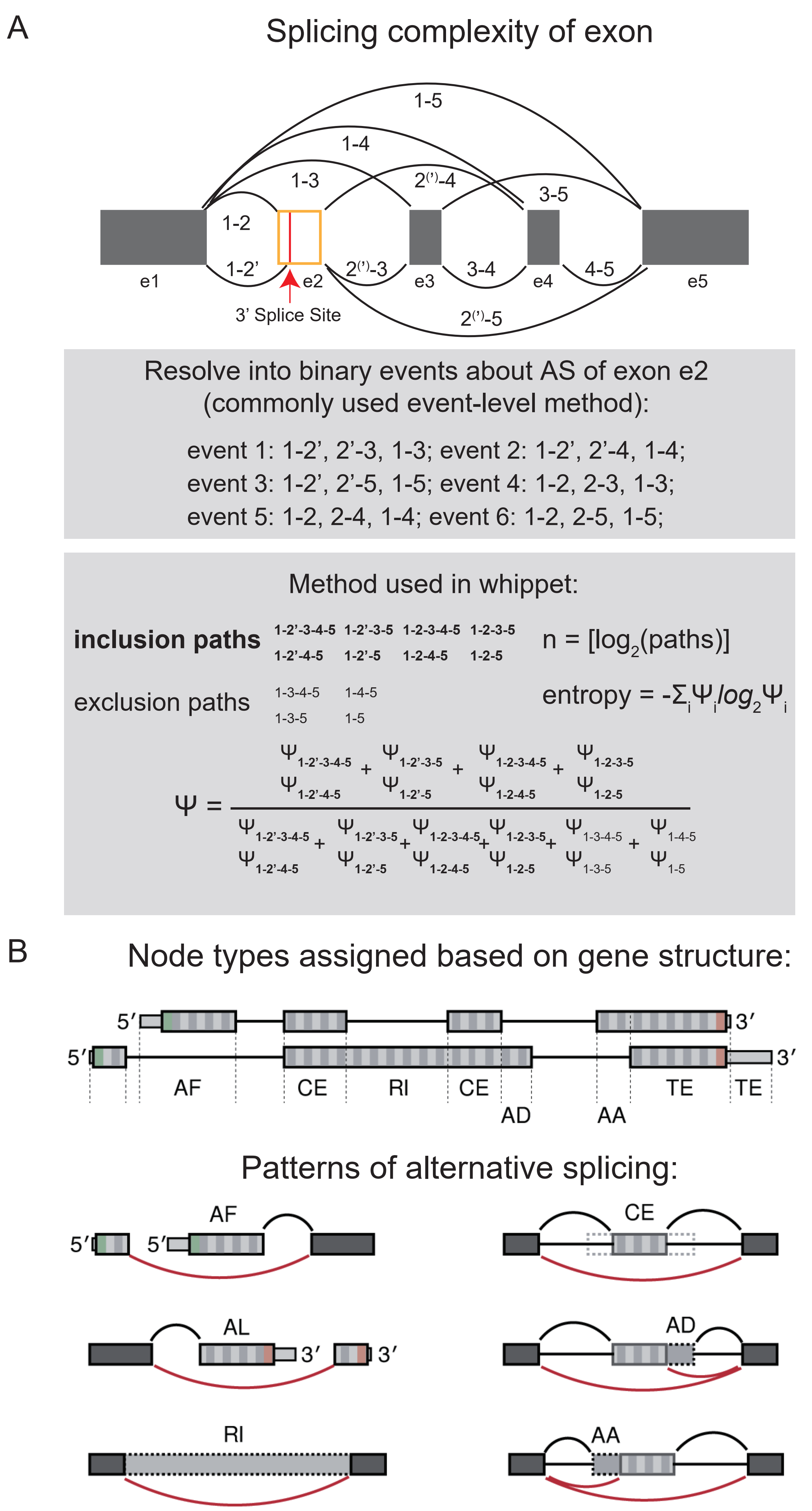


**Fig. S1** Schematic diagram for events with high splicing complexity and events definition in Whippet. (A), top: the complexity of alternative exon from a gene with 5 exons (e1:e5). e2 is an alternative exon (orange) with an extra 3’ splice sites (red arrow). The edges between exons are the connections between exons. Middle: all possible binary events are shown below according to commonly used event-level method. In whippet there is two events from e2, one AA (from left boundary of e2 to red vertical line) and one core exon (CE; e2’, from red vertical line to right boundary of e2) event. Bottom: quantification of Ψ values and splicing complexity for the CE event e2’ with K(n) and splicing entropy. Paths number is the sum of inclusion and exclusion paths numbers. Ψ_i_ is normalized relative expression of the path i which contains (inclusion paths) or skip (exclusion paths) the node e2’. (B), Node types definition in Whippet. Upper panel is an example of gene model with two isoforms. Gene structure is collapsed into non-overlapping exon intervals (nodes). Node types are assigned based on gene structure. Bottom panel is an example skipping (red) and connecting edges (black) for each node type. (B) is from https://juliapackages.com/p/whippet.

**Fig. S2** The flowchart of our analysis. Splicing complexity and Ψ values of AS events were first quantified with Whippet. The relation between splicing complexity of exons and genes’ features were explored. Features related to exons’ splicing complexity were identified by machine learning models. Dynamics of alternative exons in splicing complexity across tissues/orthologous tissues and during development were compared.





**Fig. S3** Splicing entropy distribution of alternative CE events in protein-coding genes in six tissues. The left is alternative CE events in all protein-coding genes (All CE events); the right is the alternative CE events from exons conserved in seven species (Conserved CE events).
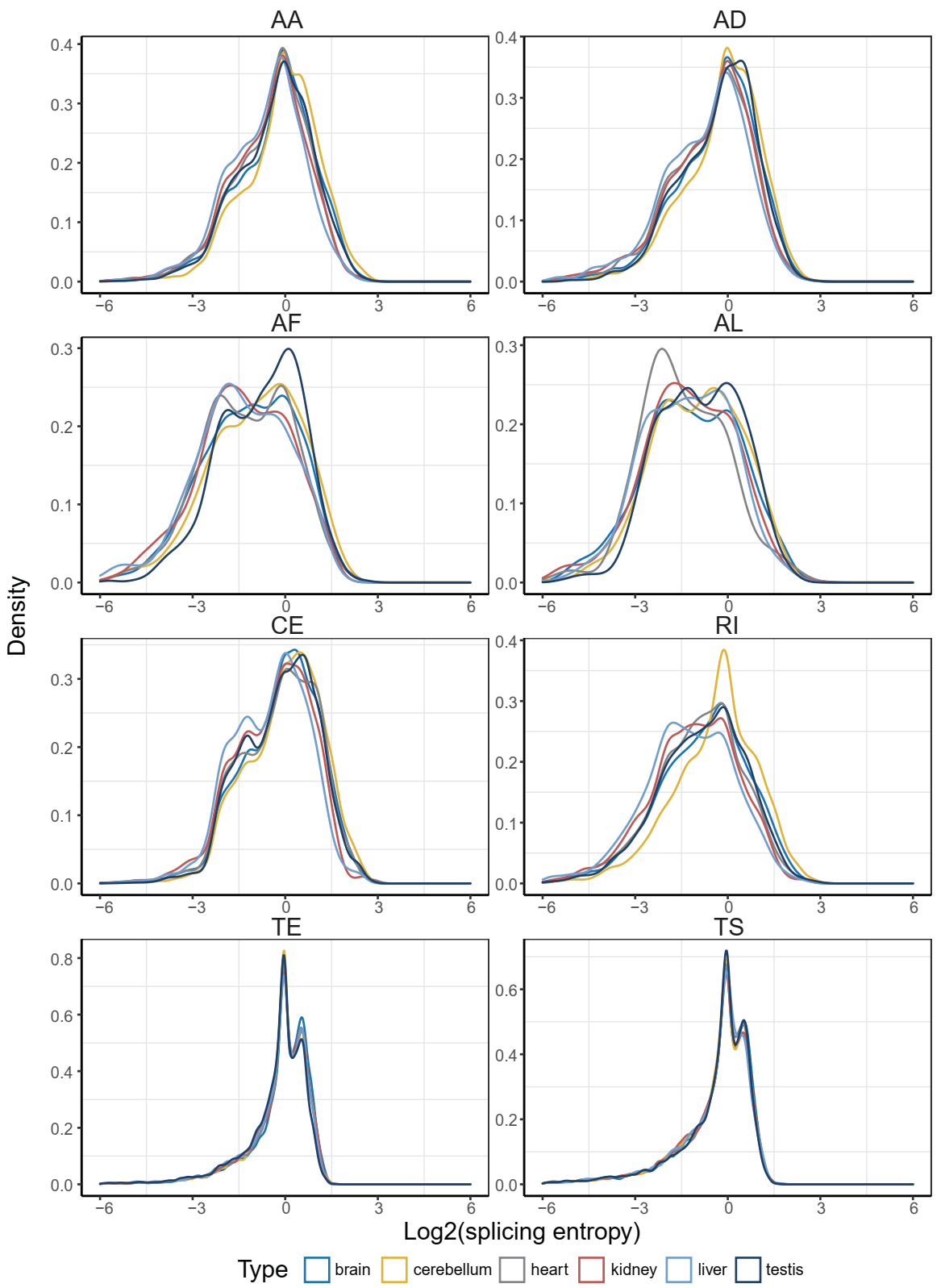


**Fig. S4** Splicing entropy distribution of all eight AS types in all six tissues of human.


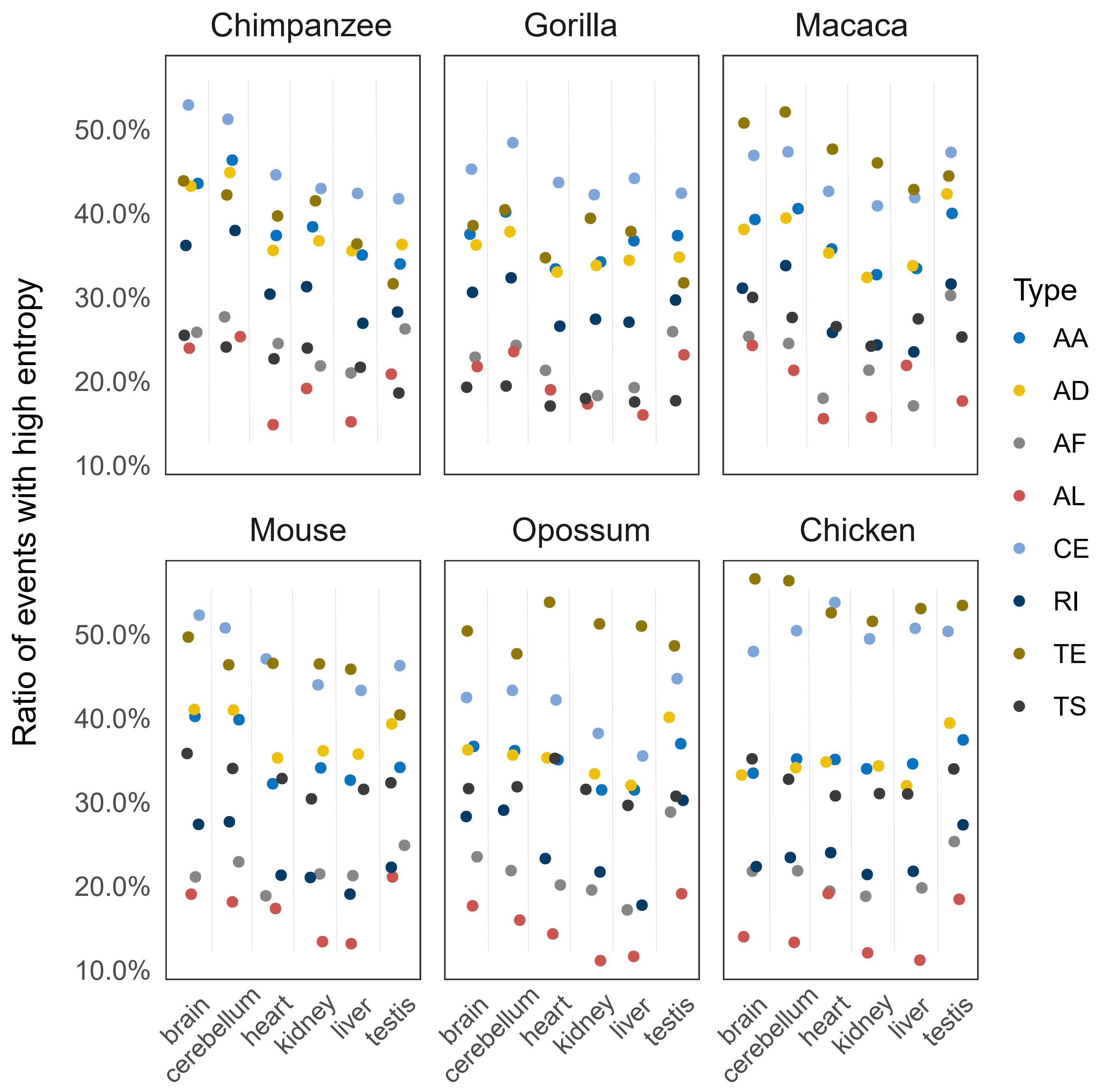


**Fig. S5** Frequencies for each type of AS events with high splicing entropy (≥ 1.0).


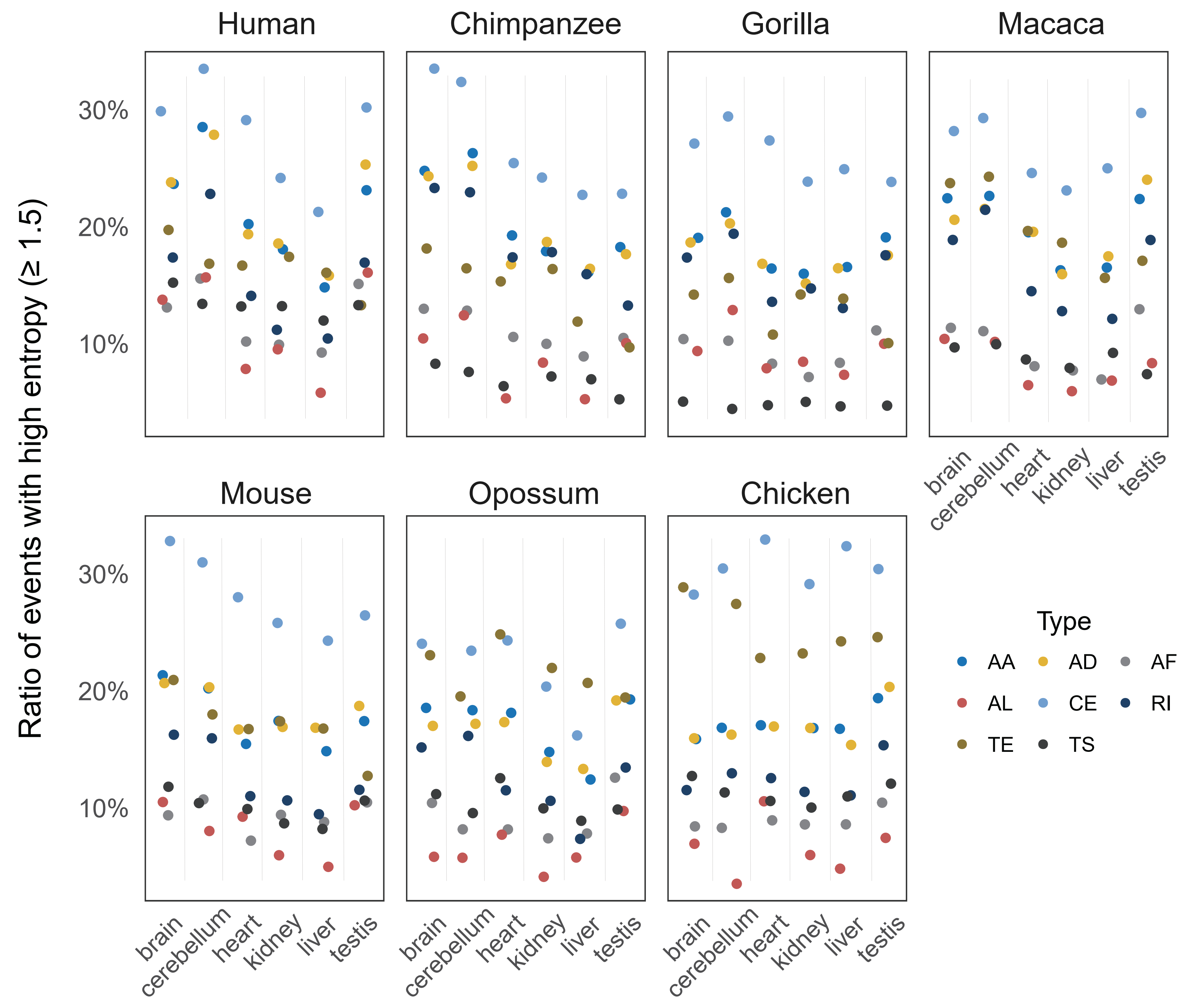


**Fig. S6** Frequencies for each type of AS events with stricter cutoff (splicing entropy ≥ 1.5).

**
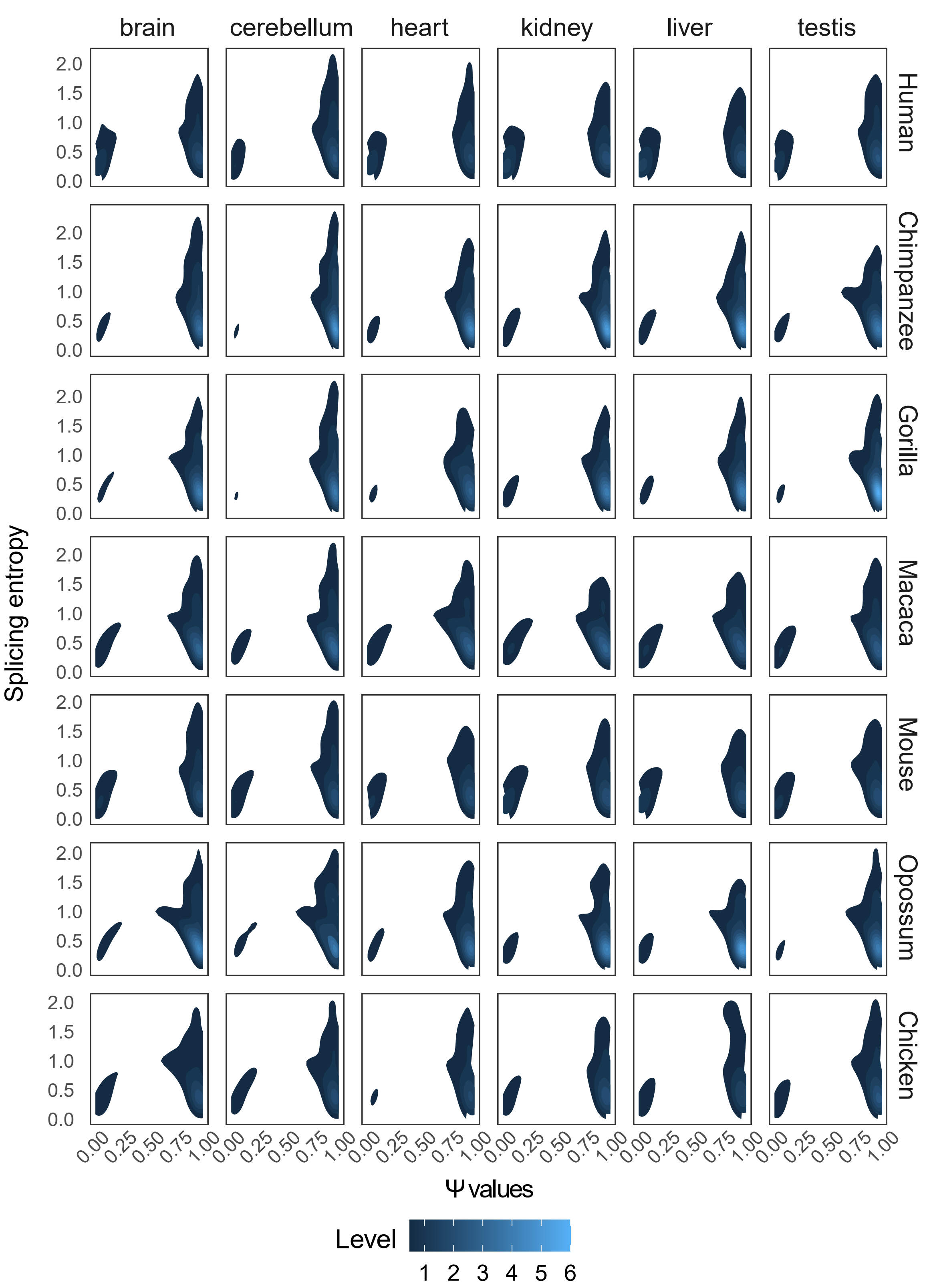
**

**Fig. S7** Density plot for Ψ values (x-axis) and splicing entropy (y-axis) across seven species.


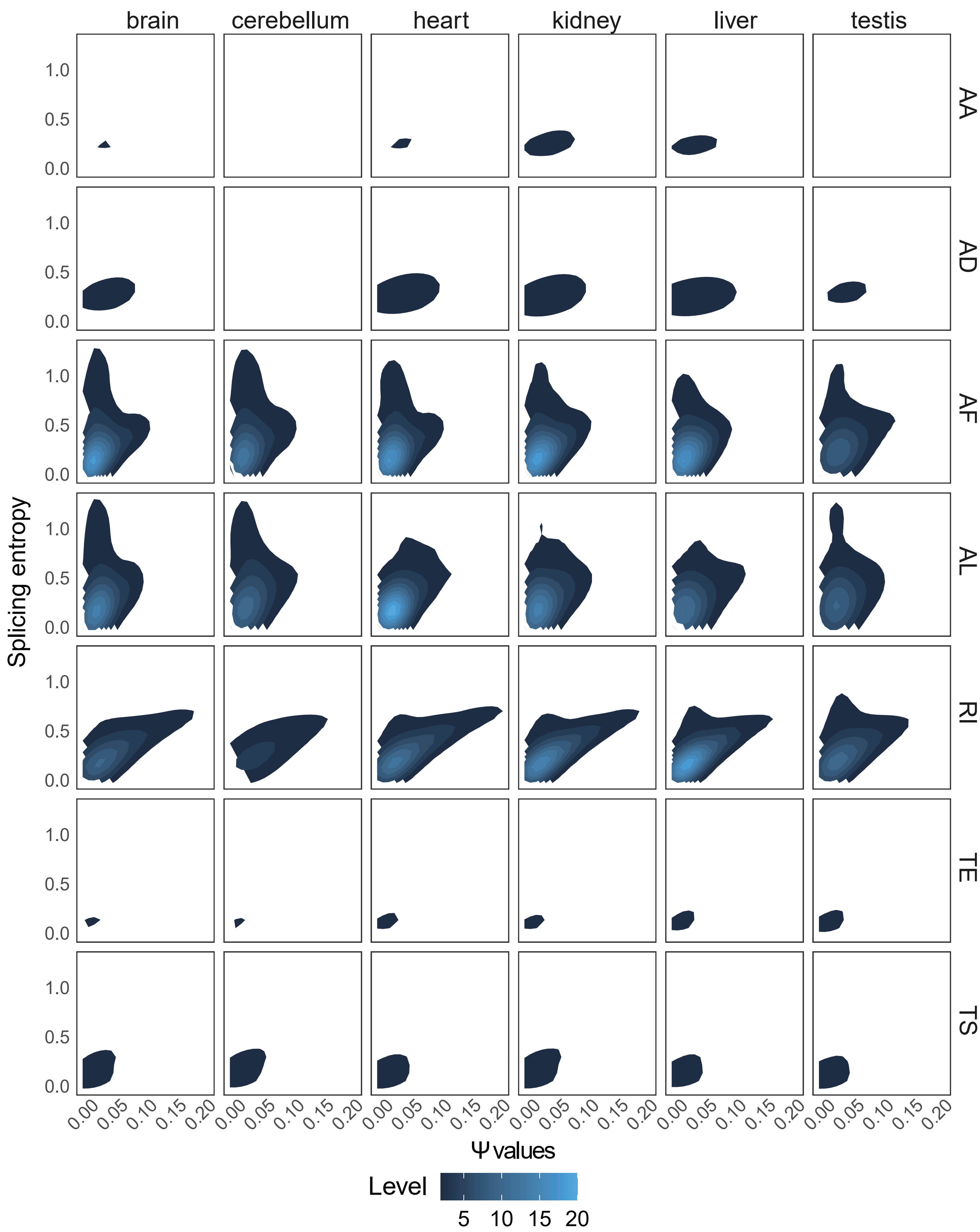


**Fig. S8** Density plot for Ψ values (x-axis) and splicing entropy (y-axis) for seven events (except for CE) in six tissues of human.

**
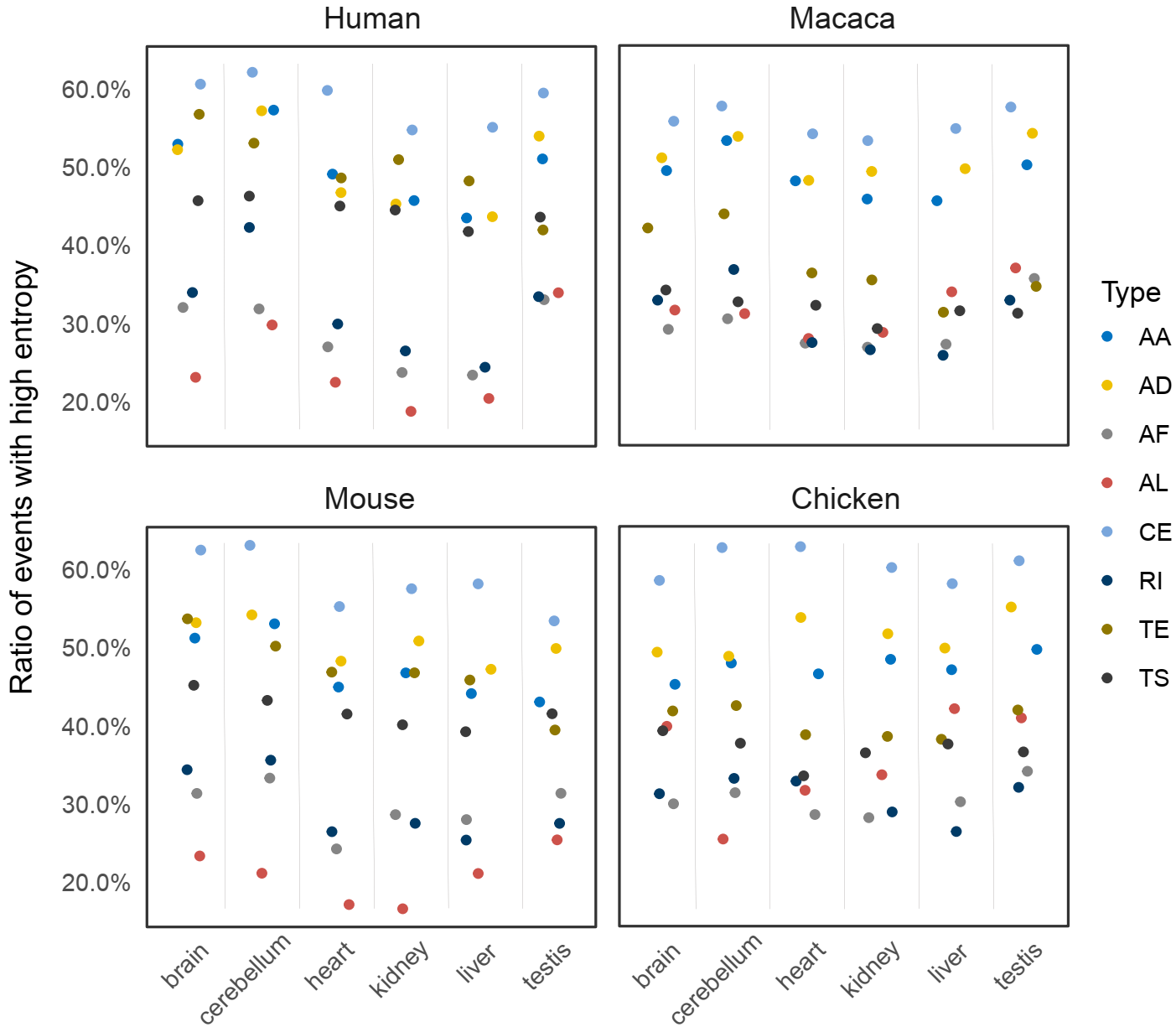
**

**Fig. S9** Frequencies for each type of AS events with high splicing entropy (≥ 1.0) according to de novo assembly transcript from Mazin et al., 2021 (Mazin PV, Khaitovich P, Cardoso-Moreira M, Kaessmann H. Alternative splicing during mammalian organ development. Nature Genetics. 2021; 53:925-+).

**
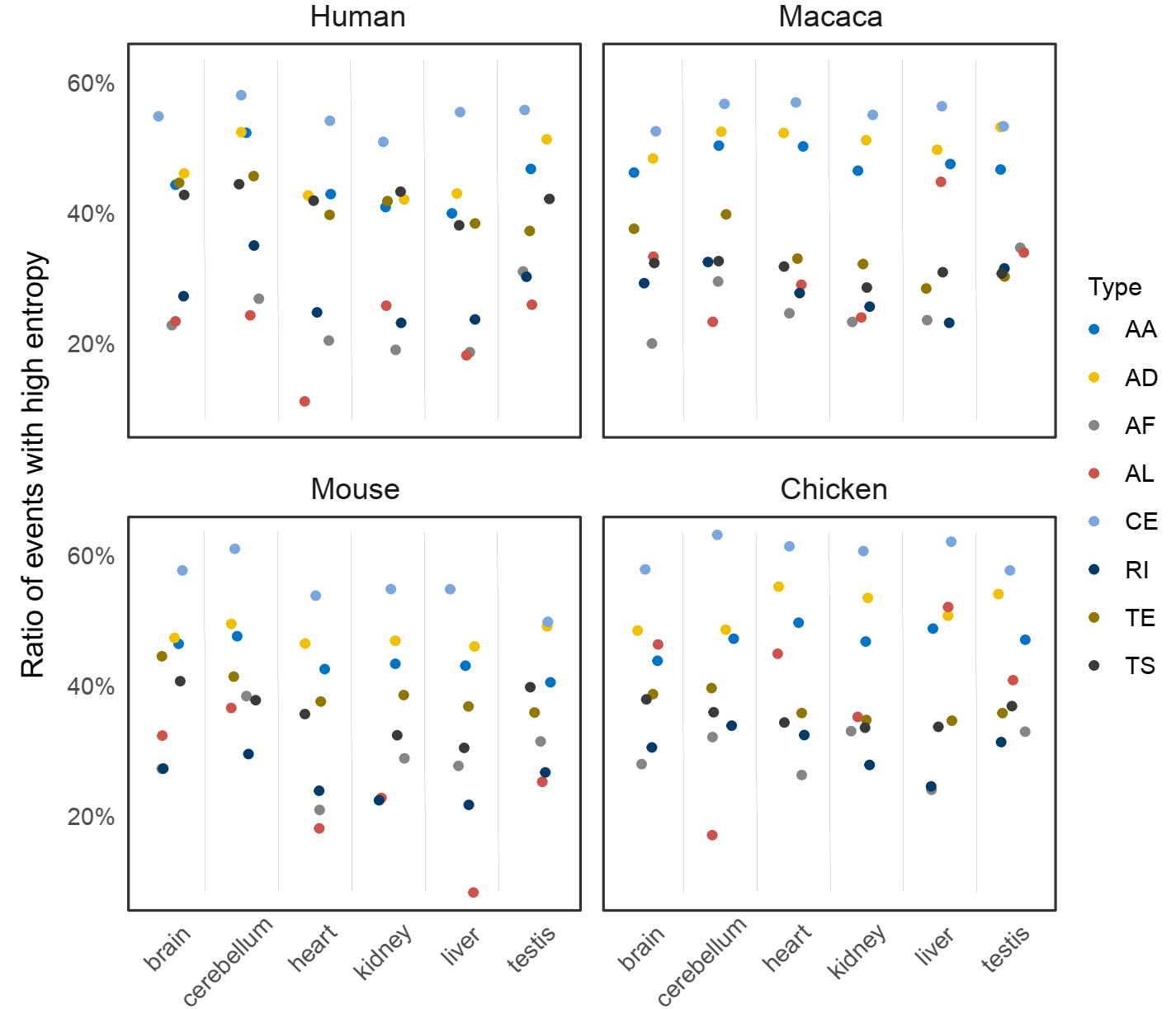
**

**Fig. S10** Frequencies for each type of AS events with high splicing entropy (≥ 1.0) after downsampling to same sequencing depth, according to de novo assembly transcript from Mazin et al., 2021 (Mazin PV, Khaitovich P, Cardoso-Moreira M, Kaessmann H. Alternative splicing during mammalian organ development. Nature Genetics. 2021; 53:925-+).

**
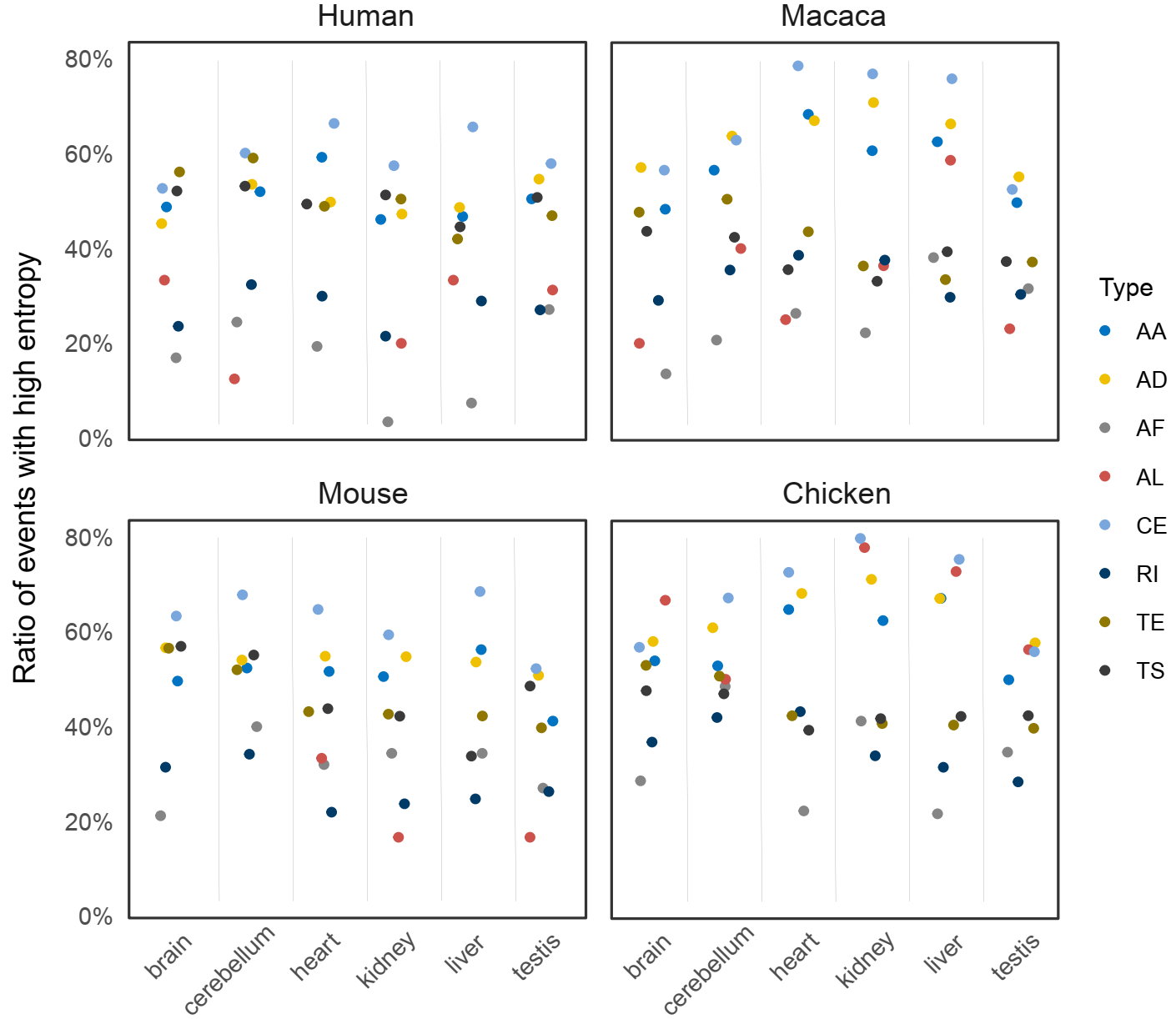
**

**Fig. S11** Frequencies for each type of AS events with high splicing entropy (≥ 1.0) in genes with high expression levels (TPM > 50 and total reads number > 50), according to de novo assembly transcripts from Mazin et al., 2021 (Mazin PV, Khaitovich P, Cardoso-Moreira M, Kaessmann H. Alternative splicing during mammalian organ development. Nature Genetics. 2021; 53:925-+).

**
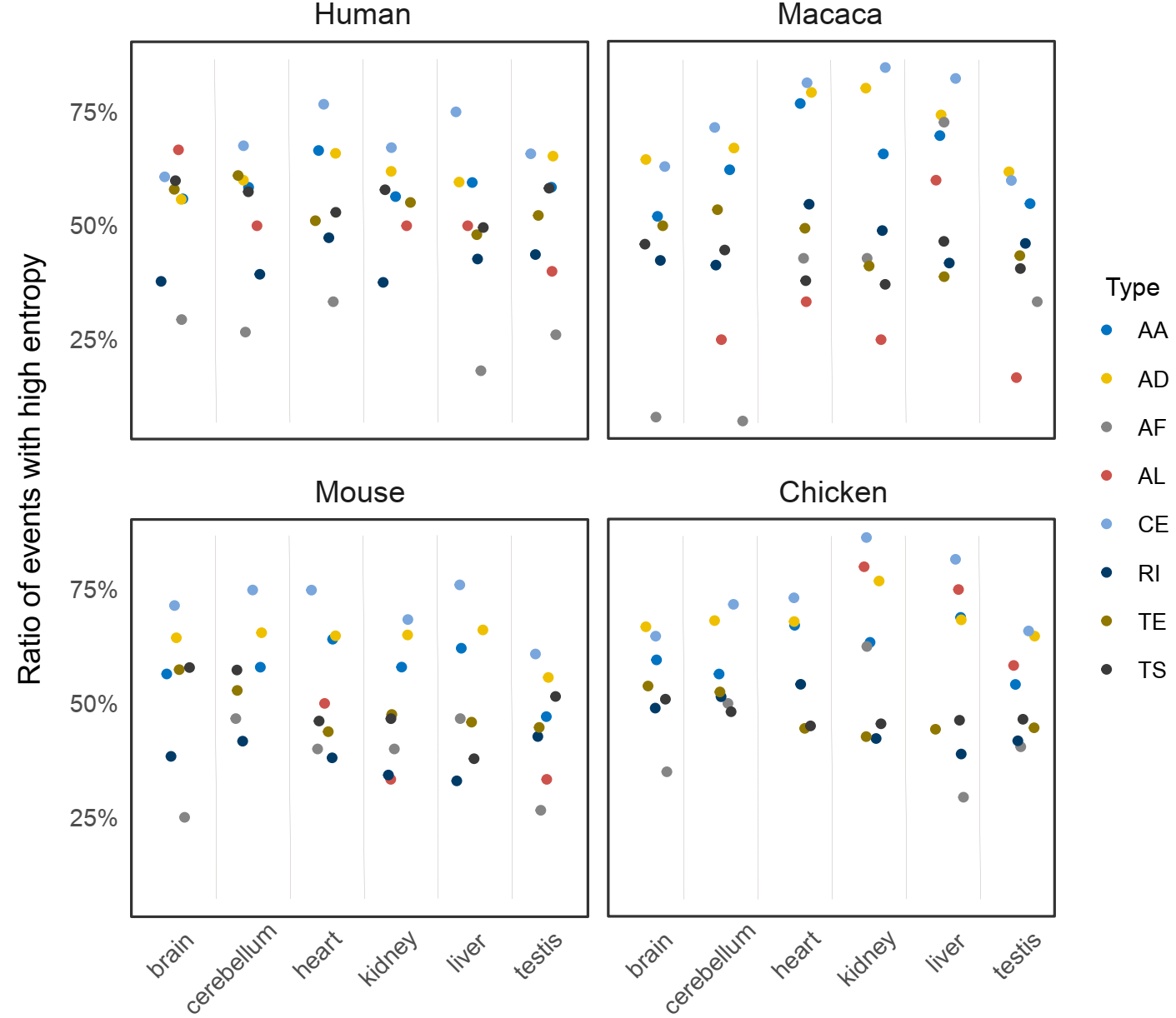
**

**Fig. S12** Frequencies for each type of AS events with high splicing entropy (≥ 1.0) using 0.05 < Ψ < 0.95 as cut-off, according to de novo assembly transcript from Mazin et al., 2021 (Mazin PV, Khaitovich P, Cardoso-Moreira M, Kaessmann H. Alternative splicing during mammalian organ development. Nature Genetics. 2021; 53:925-+).

**
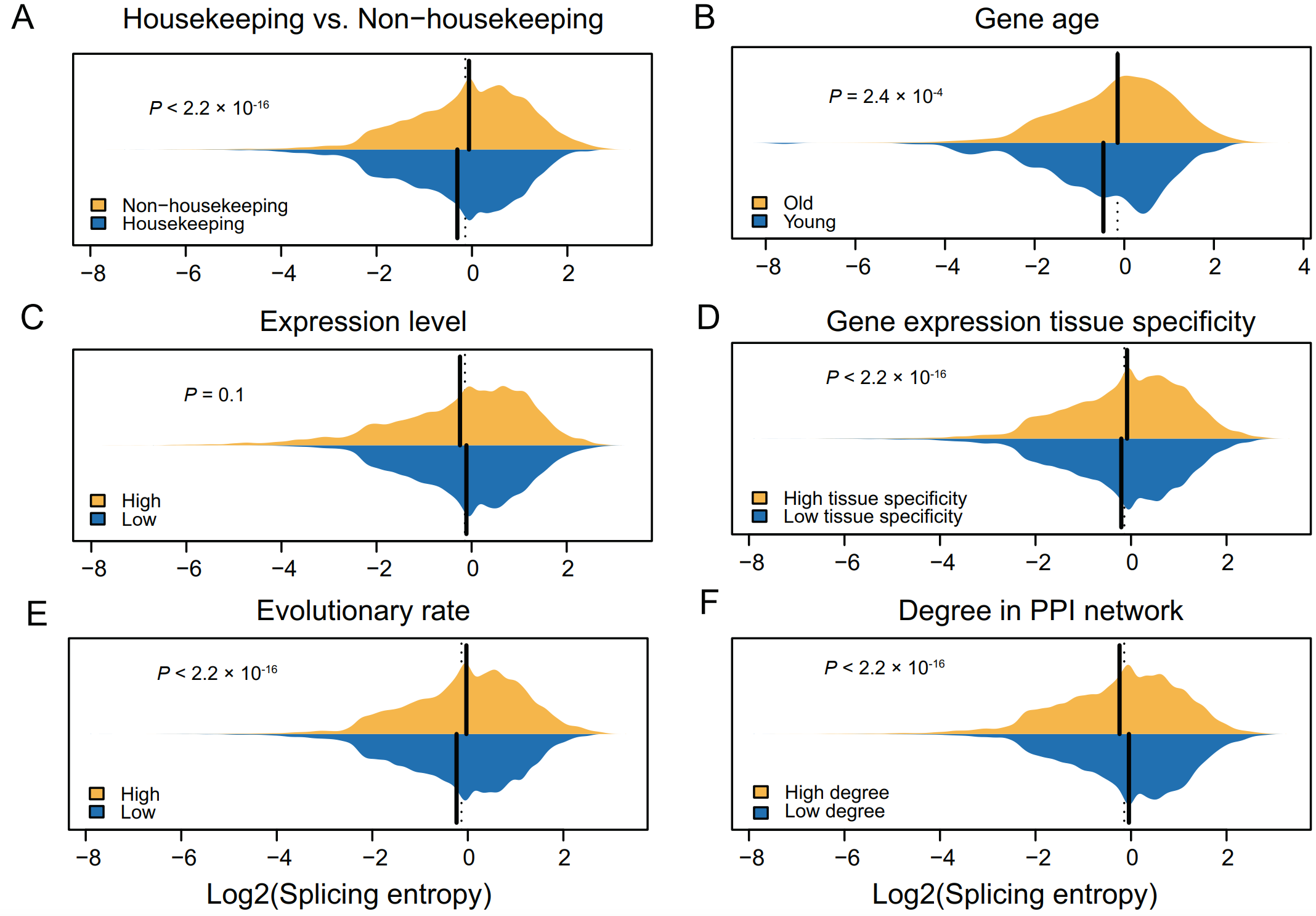
**

**Fig. S13** The bean charts display differential splicing entropy of alternative CE events in genes with expression level larger than 10 TPM. All significances were evaluated with Wilcoxon rank-sum test.

**
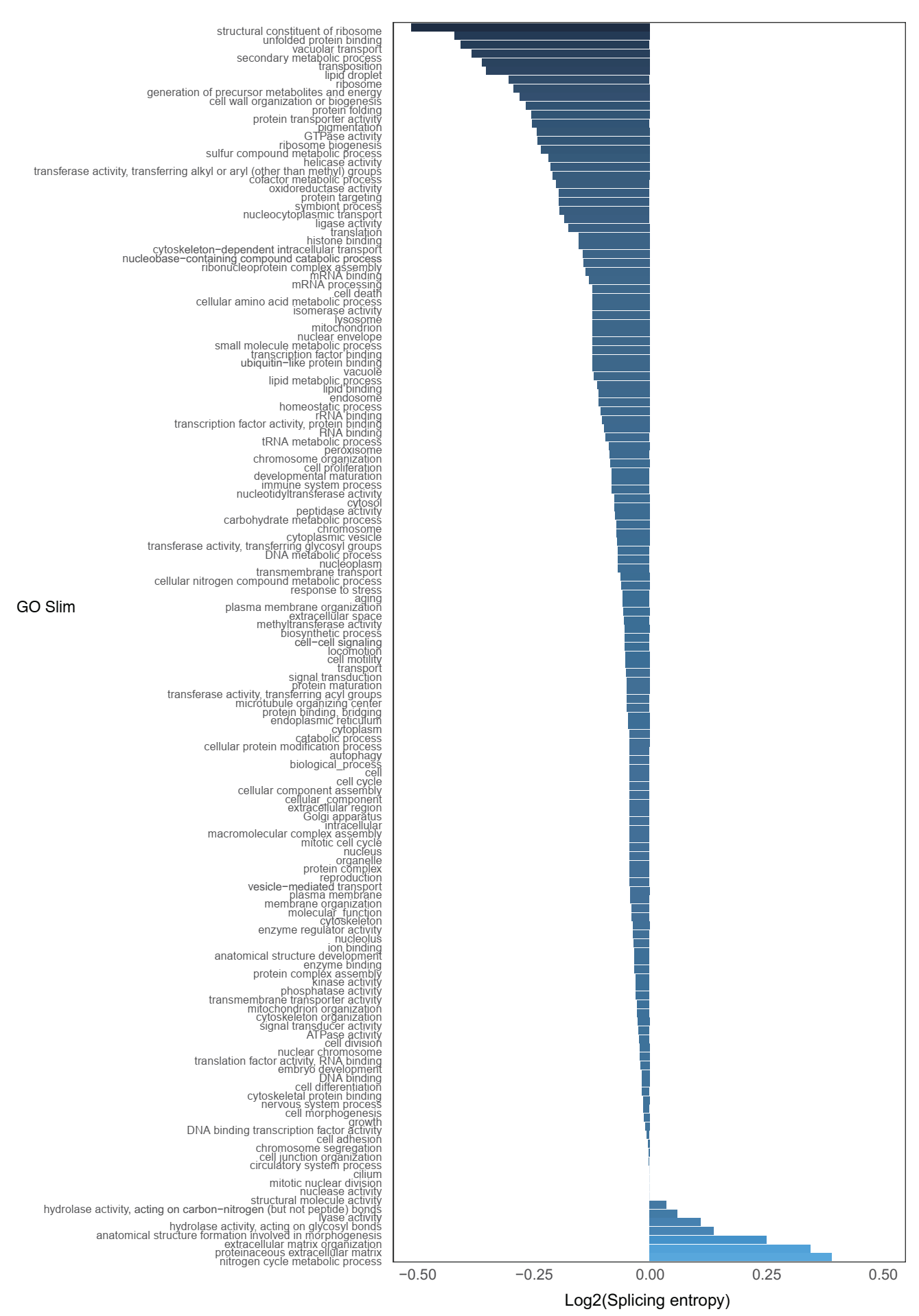
**

**Fig. S14** Rank of mean splicing entropy for genes from each GO slim, only genes that include alternative CE events were considered.

**
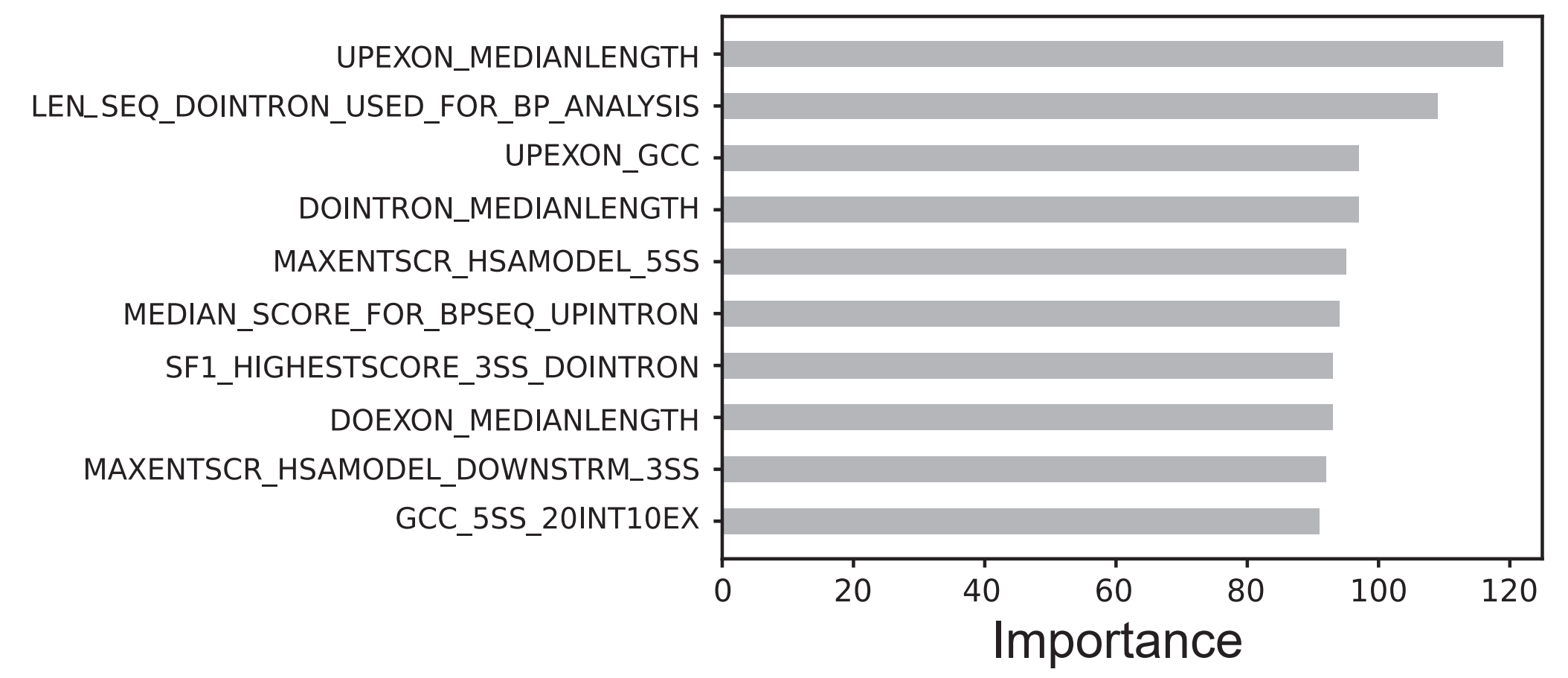
**

**Fig. S15** The feature importance for predicting splicing entropy (top 10) with deep learning neural network model.


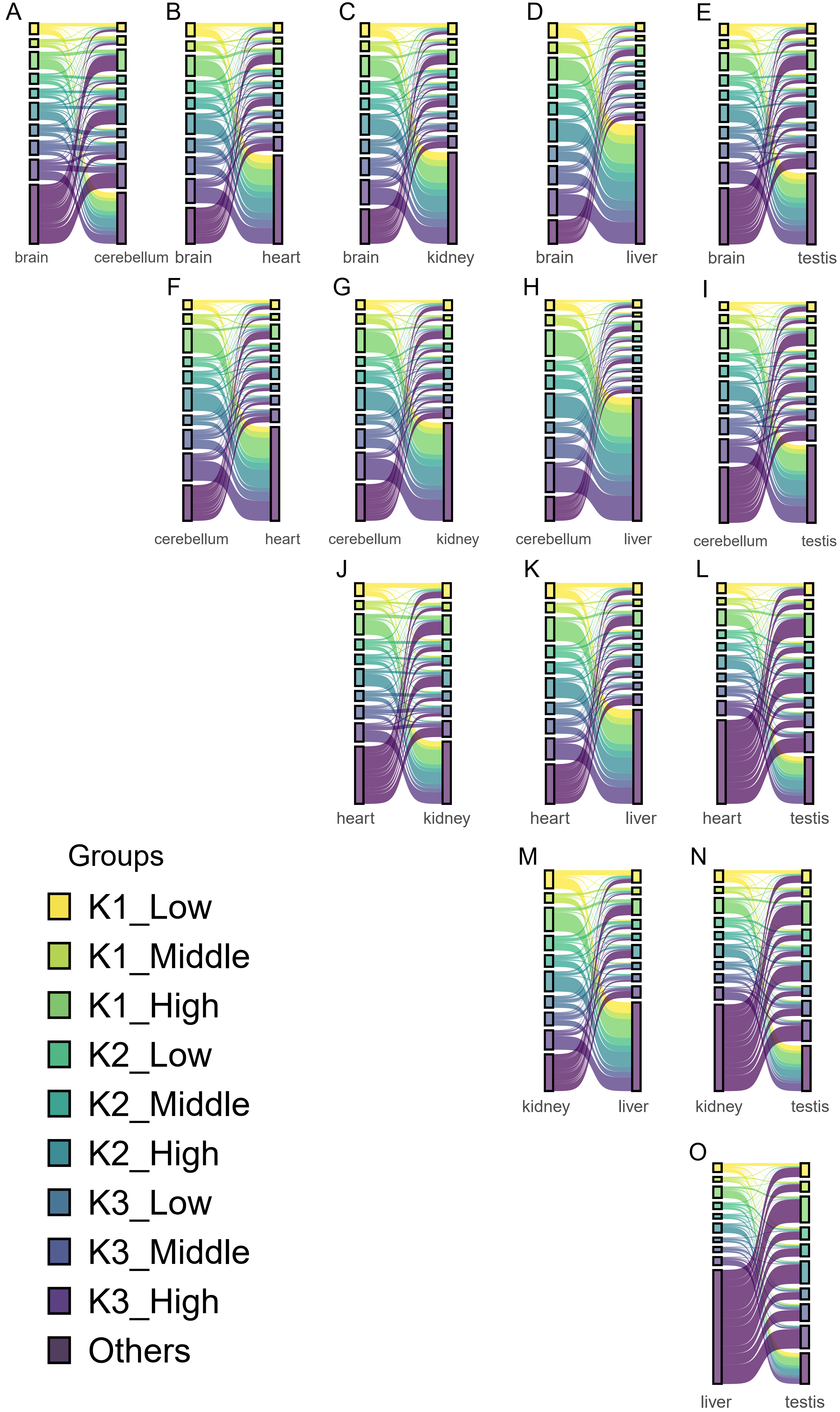


**Fig. S16** Sankey plots show conversion of events’ belonged groups among human tissues with transcripts annotation from Ensembl database.

**
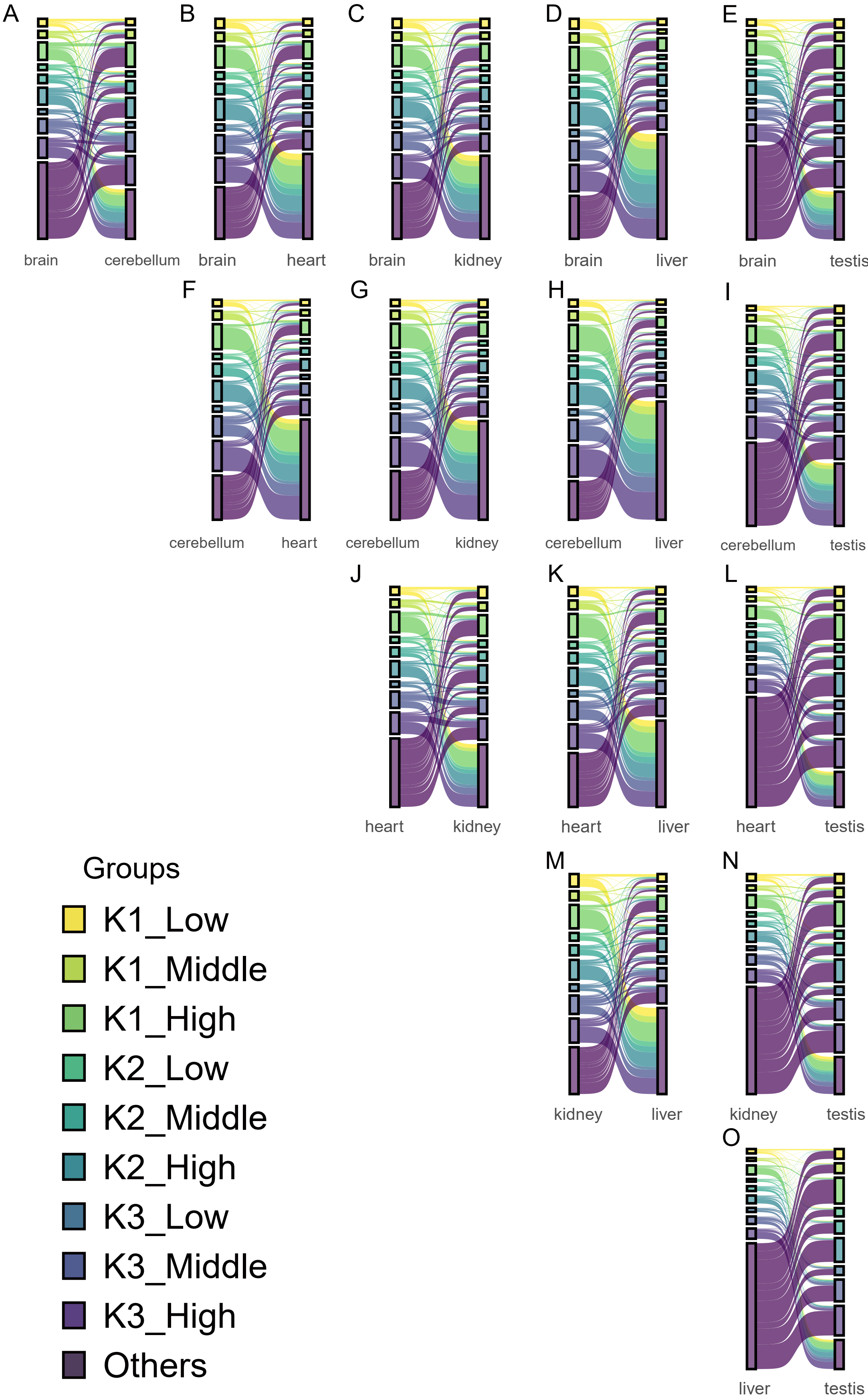
**

**Fig. S17** Sankey plots show conversion of events’ belonged groups among human tissues after downsampling with de novo assembled transcripts annotation.

**
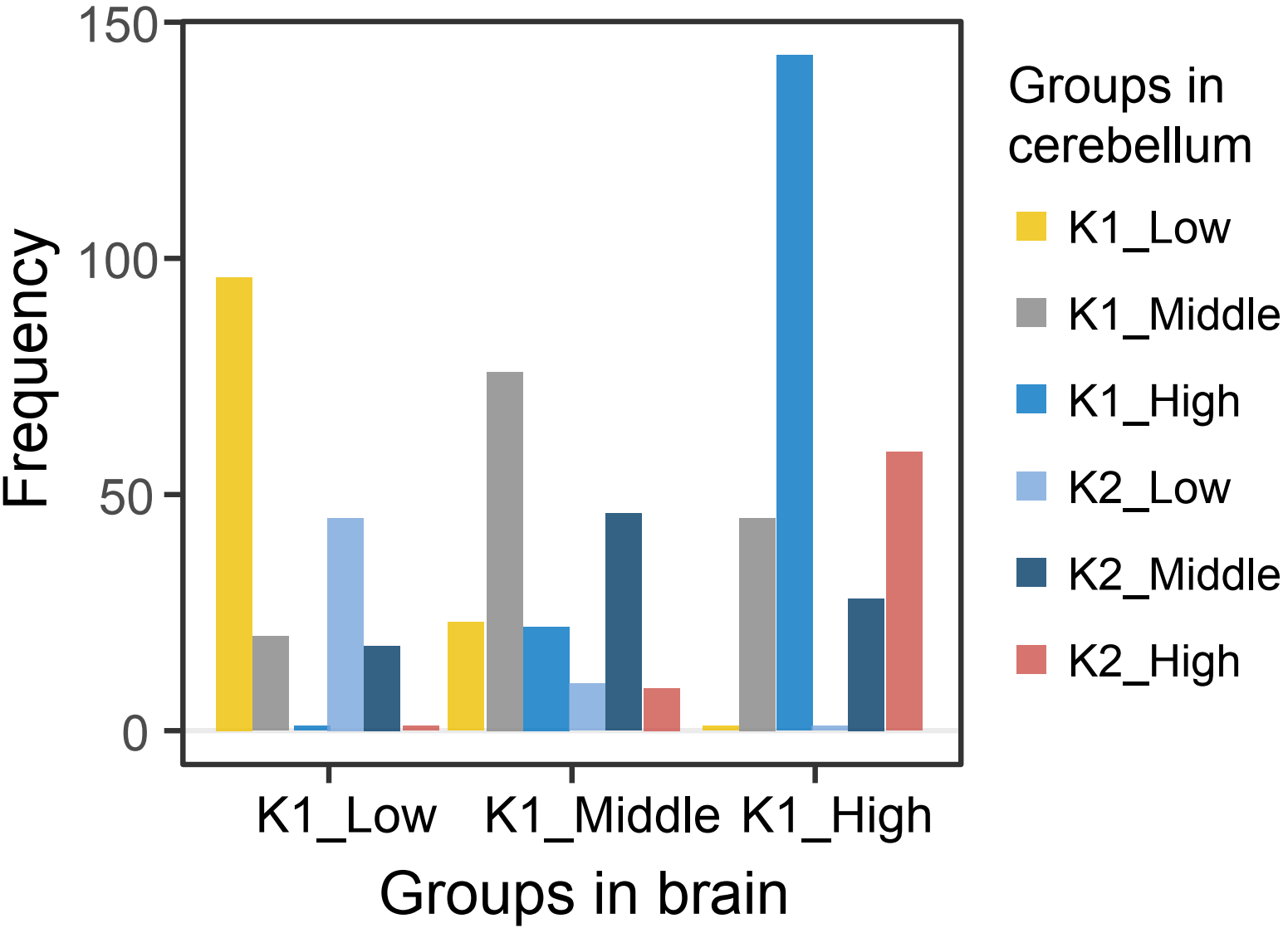
**

**Fig. S18** Bar plot showing the frequencies of CE events from different groups in brain and cerebellum with de novo assembled transcripts.


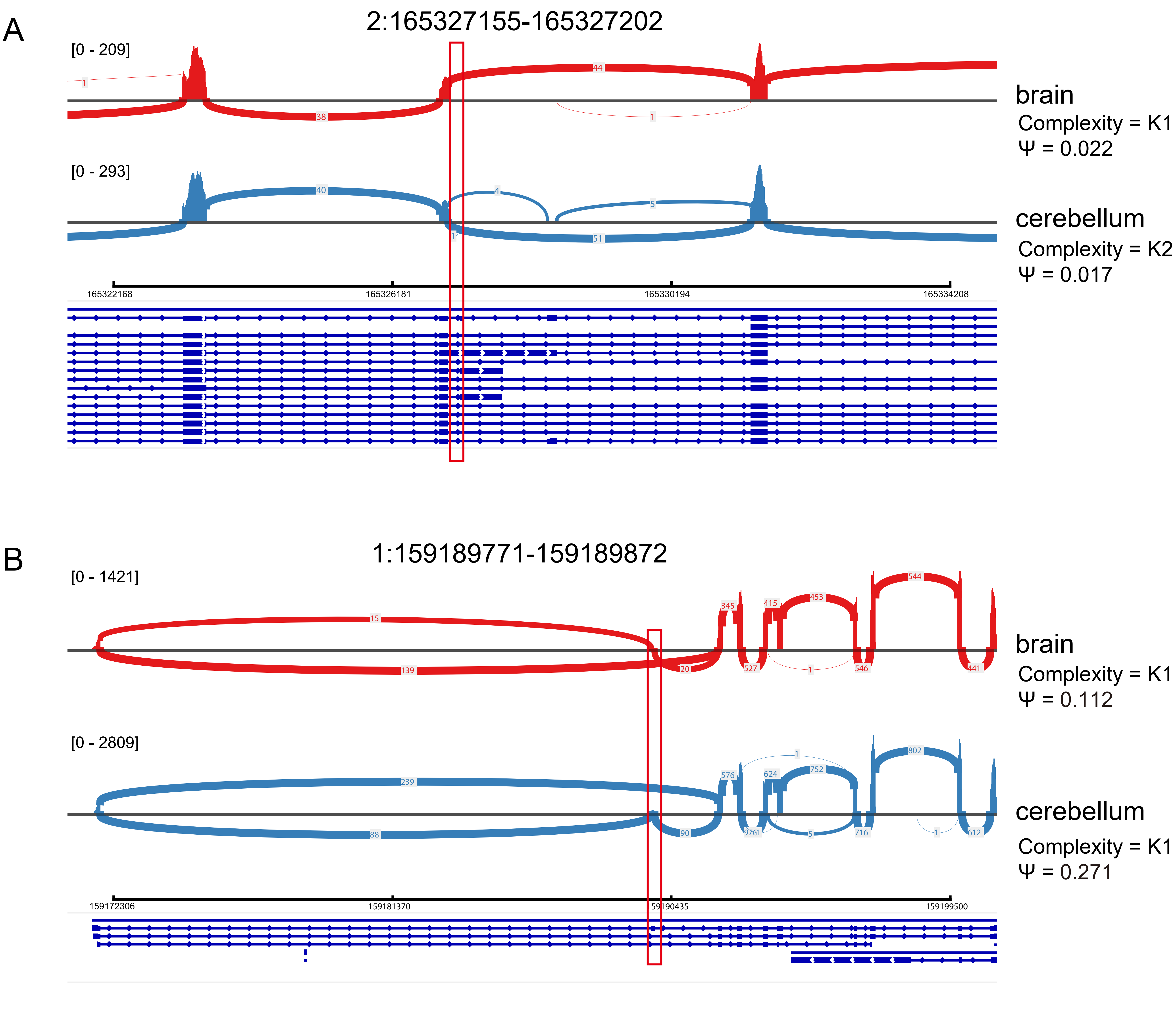


**Fig. S19** Integrative Genomics Viewer (IGV) shows the read density for one event (﻿2:165,327,155-165,327,202) that have higher complexity in cerebellum (A) and another event (﻿1:159,189,771-159,189,872) that have higher Ψ value in cerebellum relative to brain (B). Red brackets show the sites of target exons.


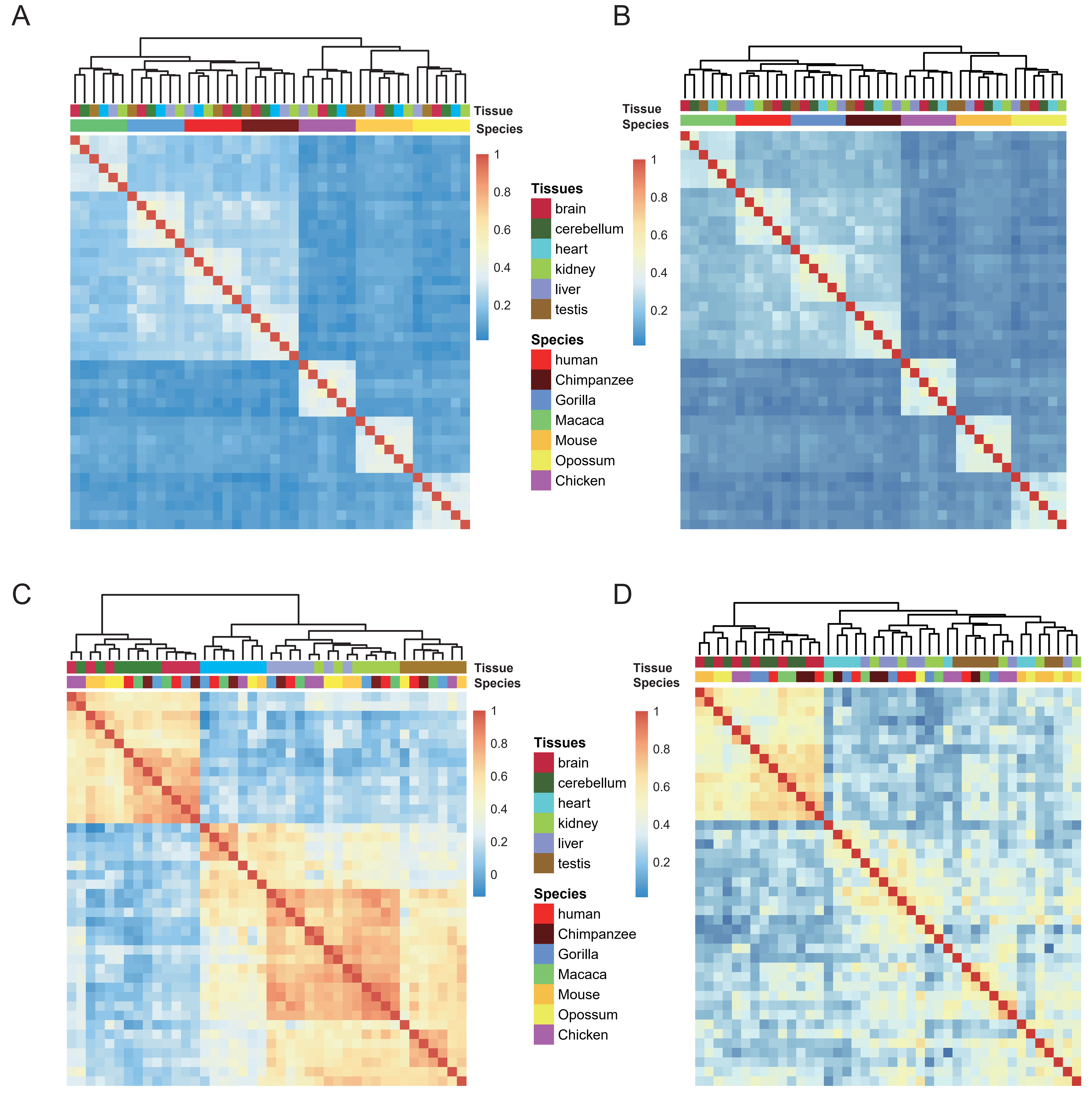


**Fig. S20** Clustering for splicing entropy and Ψ values. (A), Hierarchical clustering for Ψ values for 22,294 orthologous exons which are alternative spliced in any species in 5,255 genes. (B), Hierarchical clustering with splicing entropy using events in (A). (C), Hierarchical clustering for Ψ values of 251 conserved CE exons in 205 genes. (D), Hierarchical clustering with splicing entropy using events in (C). Cluster distance: Euclidean; cluster method: complete. Colored bars above the heatmap indicate the tissues and organism. Darker red represents stronger correlation; darker blue represents weaker or no correlation.

**
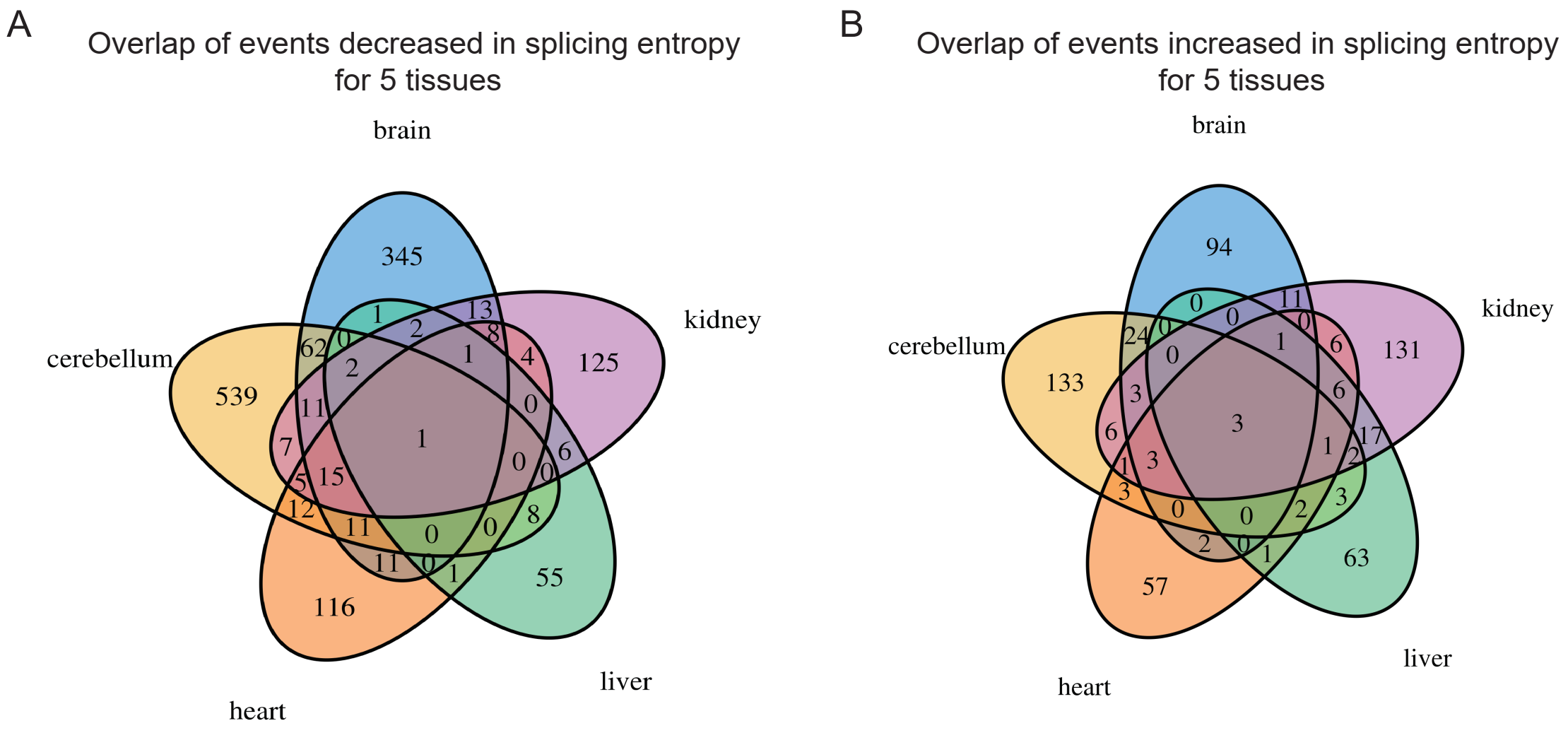
**

**Fig. S21** Venn diagram showing overlap of CE events with decreased (A)/increased (B) splicing entropy during evolution among tissues.

**
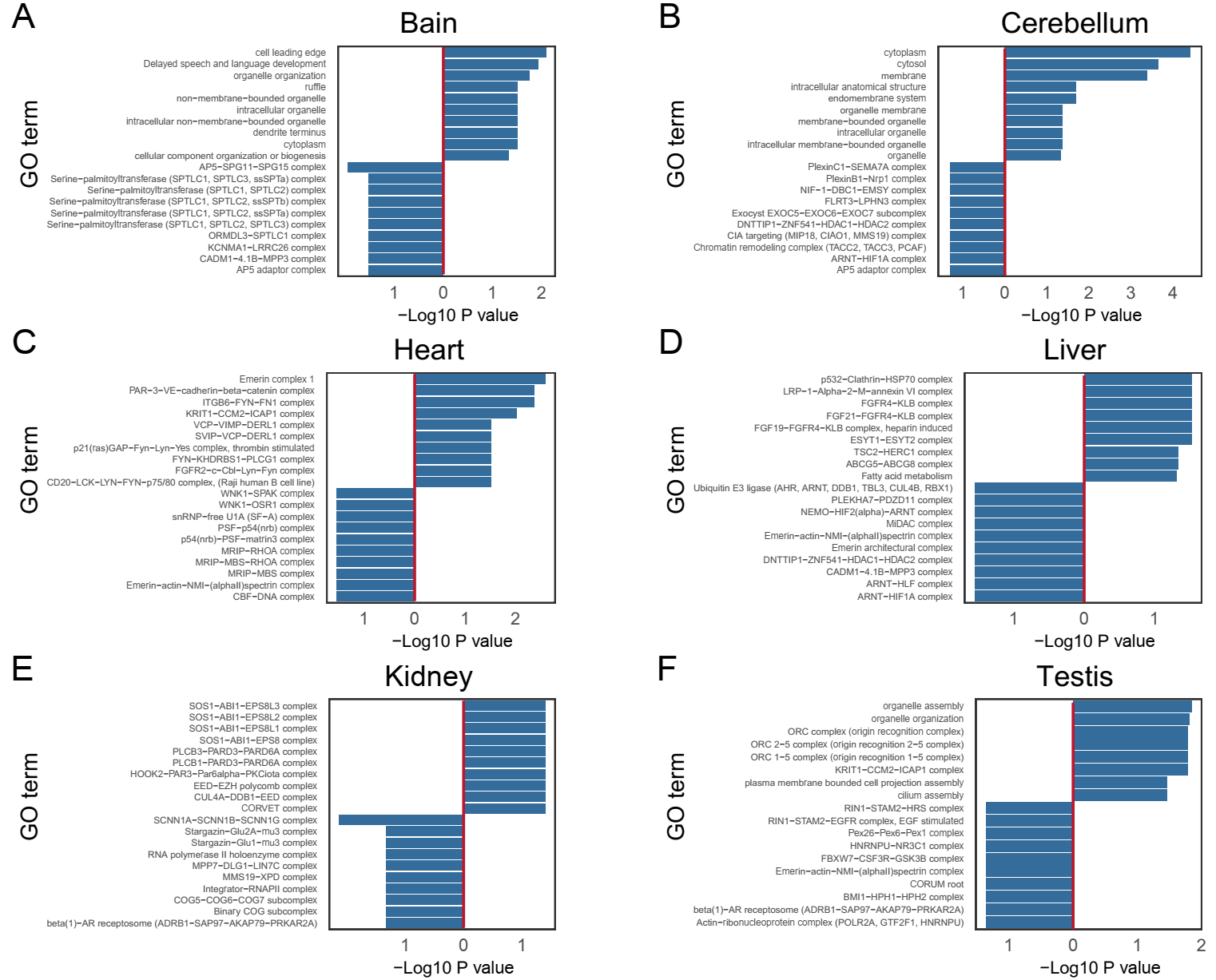
**

**Fig. S22** GO slim enrichment of genes including CE events with decreased/increased splicing entropy during evolution. (A) to (F), Bar plot displaying the enriched GO for decreased events in splicing complexity for six tissues. The left of the middle red line are enriched GO terms with events decreased in splicing entropy, the right is for enriched GO terms with events increased in splicing entropy during evolution.

**
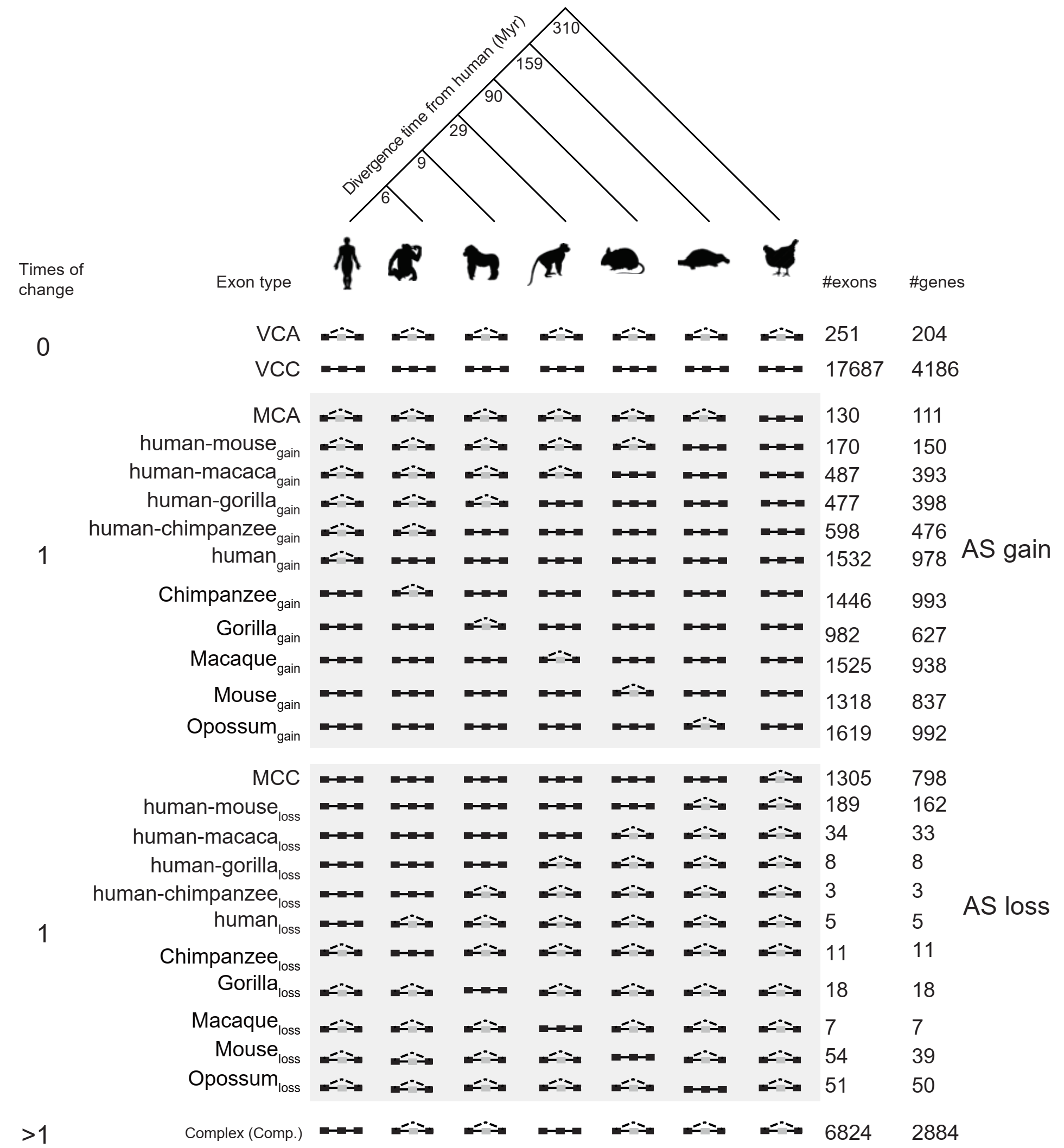
**

**Fig. S23** Phylogenetic tree of analyzed species with distance from human in millions of years (Myr). The numbers and splicing pattern for AS gain, AS loss and species-specific changes were shown left. For each splicing event group, splicing patterns and the numbers of events detected and located genes are shown.

**
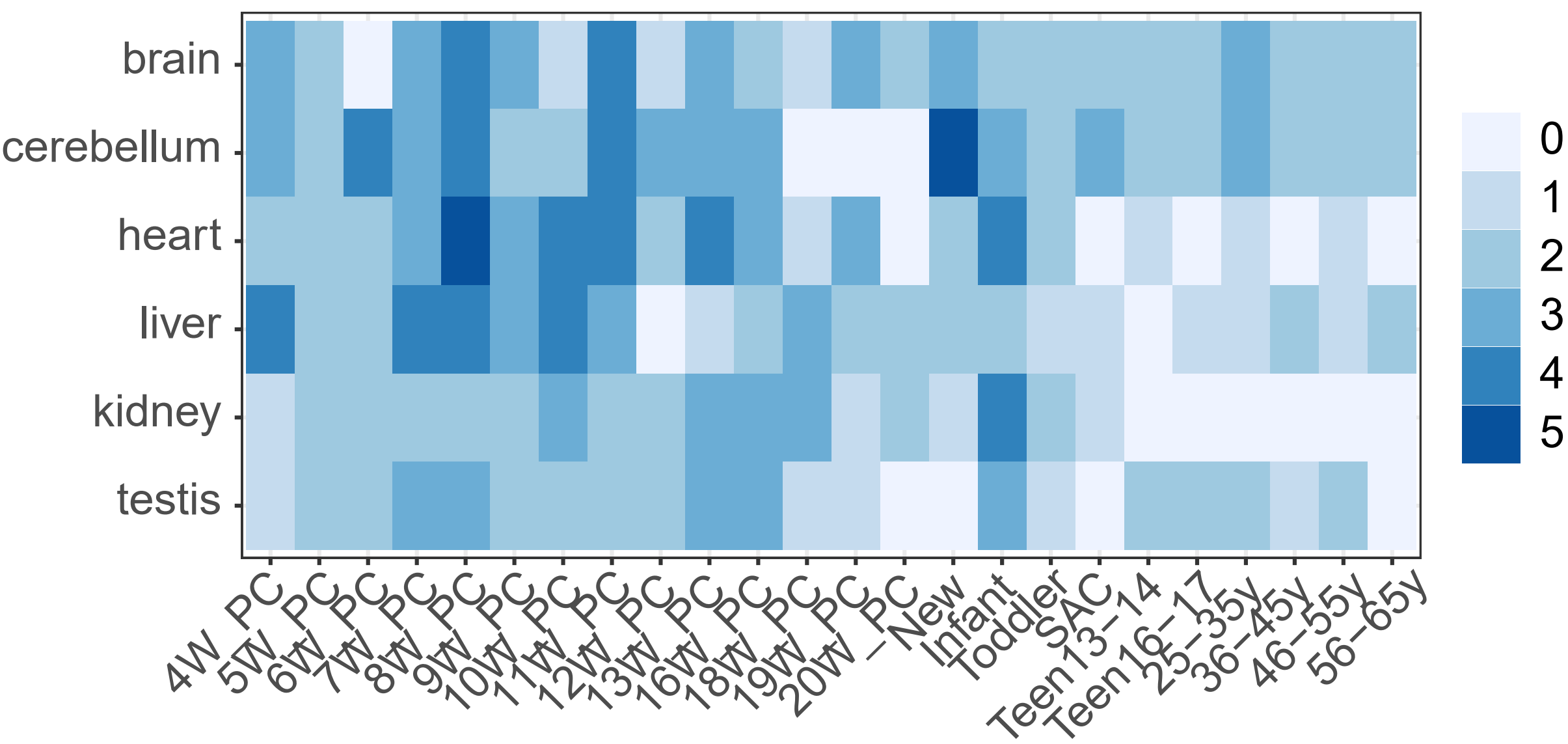
**

**Fig. S24** The numbers of biological replicates for each developmental stage across six tissues.





**Fig. S25** Hierarchical clustering for splicing entropy for CE events (change in splicing entropy larger than 1.0 at least five stages compared with the first stage) in each tissue.

**
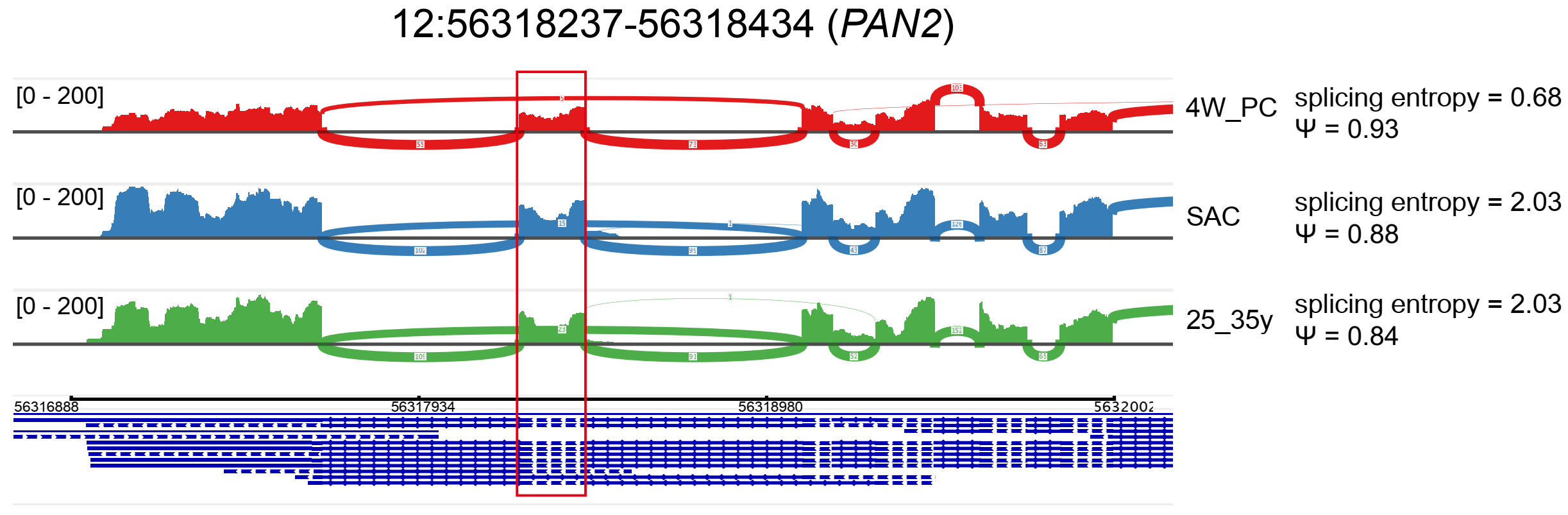
**

**Fig. S26** Integrative genomics viewer (IGV) shows the read density for one event (﻿12:56,318,237-56,318,434) in gene *PAN2* that have higher complexity in SAC and 25_35y stages. Red bracket marks target exons.


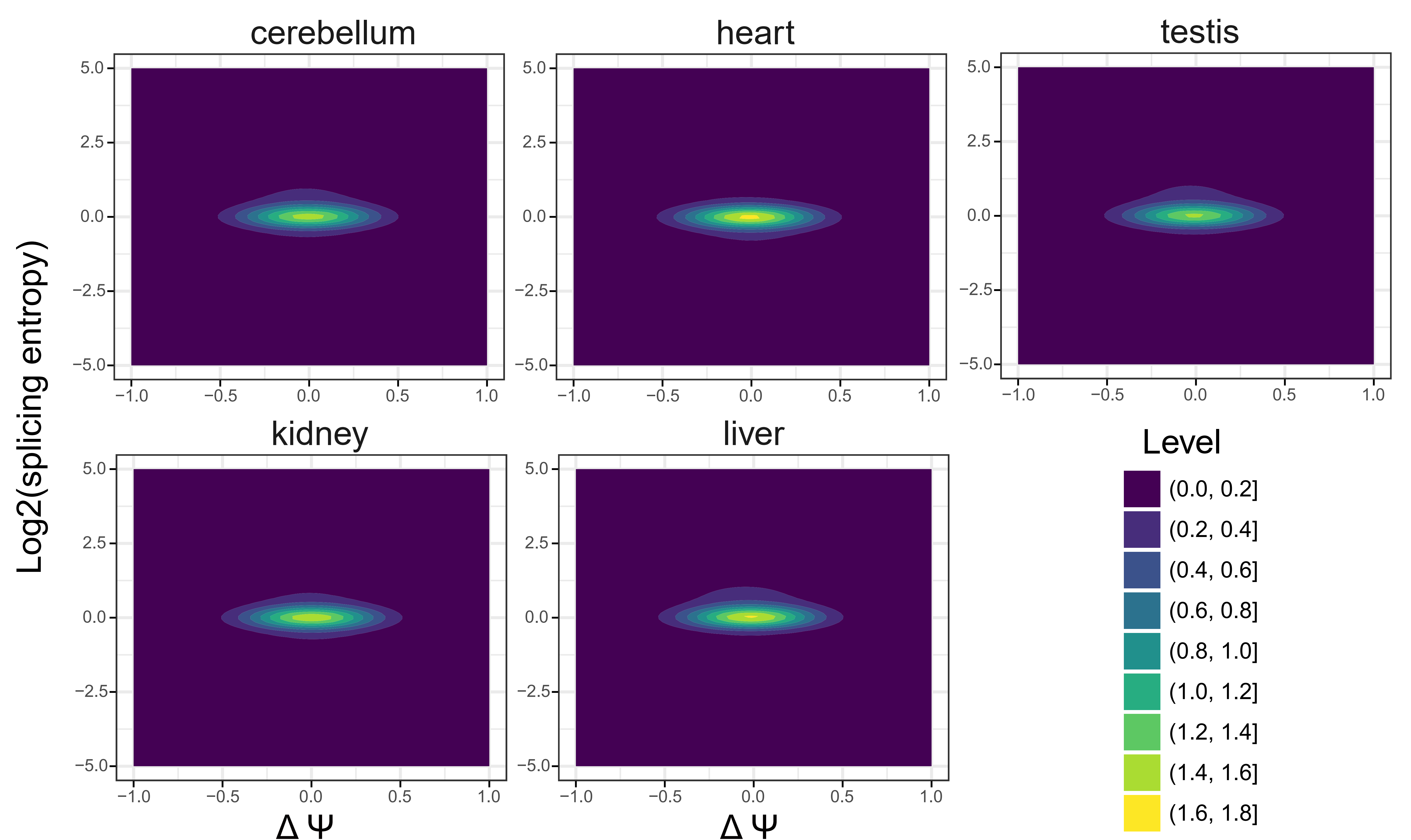


**Fig. S27** 2D kernel density plots showing the largest change in splicing entropy (y-axis) and Ψ values (x-axis) for each tissue.


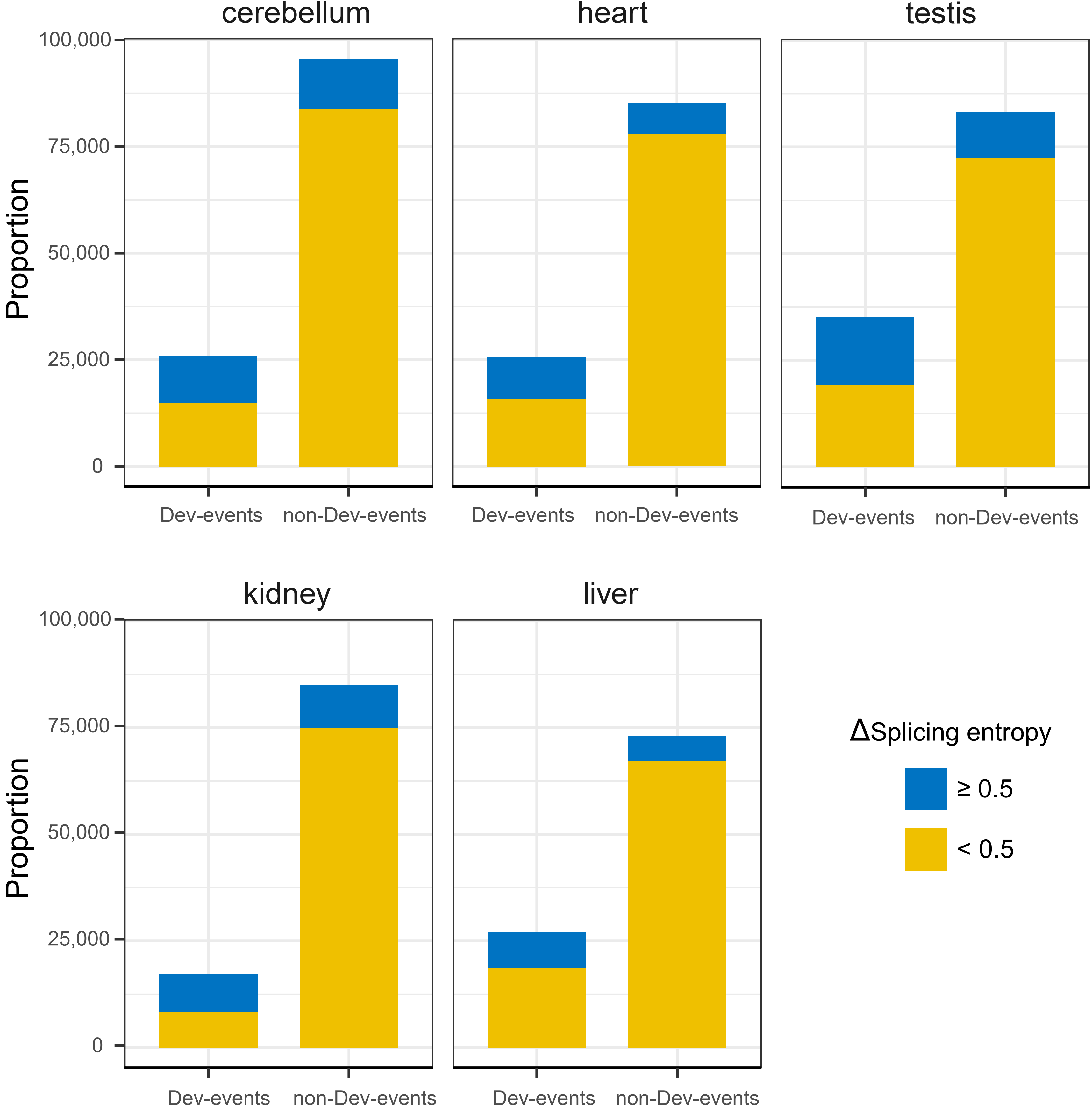


**Fig. S28** The number of events that change in splicing entropy for each tissue. Dev-events: CE events with largest change in Ψ values larger than 0.1, non-Dev-events: CE events with largest change in Ψ values less than 0.1 during development.


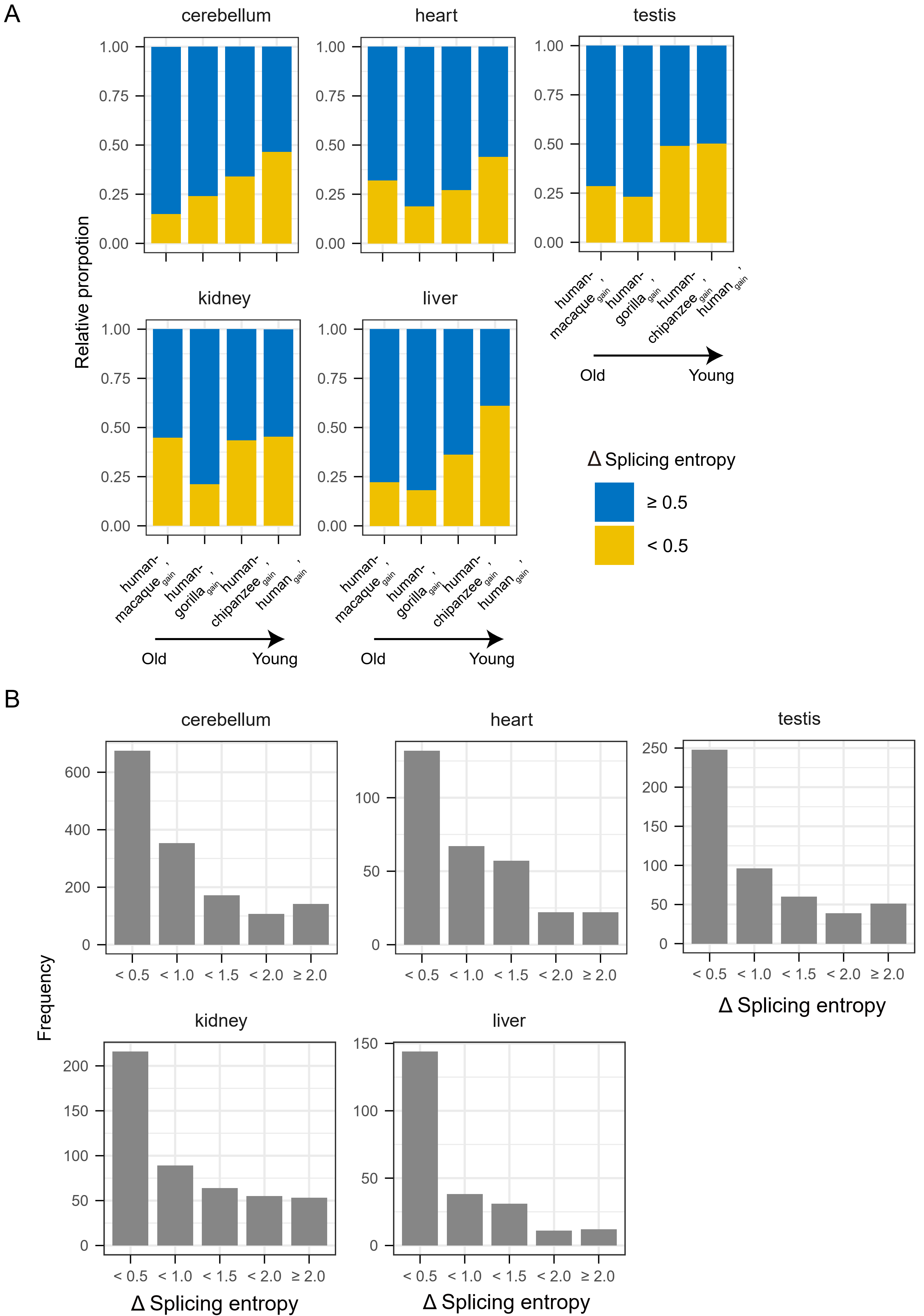


**Fig. S29** Dynamics of splicing entropy during development. **(**A), The relative ratio of events with splicing entropy changes larger than 0.5 among events that are not regulated in Ψ (max Δ Ψ < 0.1) in each age group. (B), the distribution of splicing entropy changes for non-Dev-events events in human_gain_’ group for each tissue.


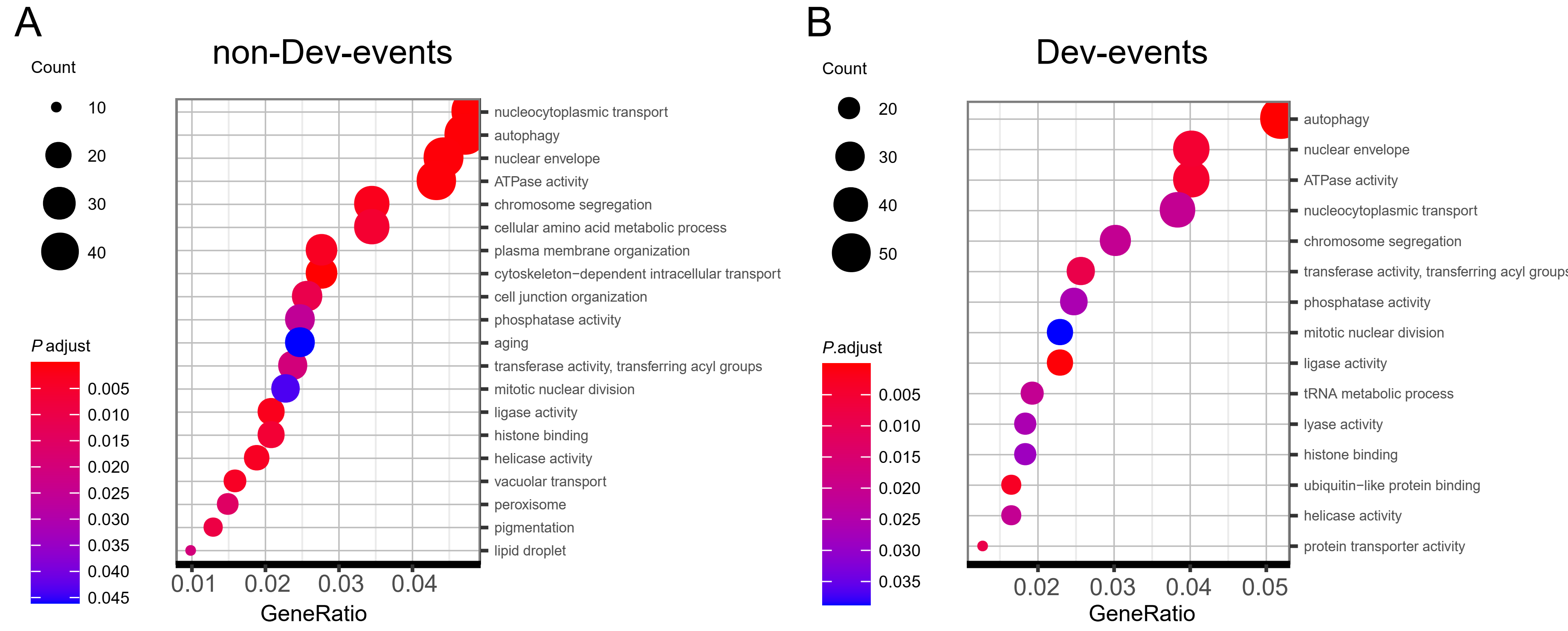


**Fig. S30** The function difference of genes with events that have different splicing entropy change during development. (A), GO and KEGG (Kanehisa, M. and Goto, S.; KEGG: Kyoto Encyclopedia of Genes and Genomes. Nucleic Acids Res. 2000; 28:27-30.) pathway enrichment for genes with non-Dev-events from human_gain_’ group. (B), Similar to (A), but for genes with Dev-events from human_gain_’ group.
